# Host genotype and time dependent antigen presentation of viral peptides: predictions from theory

**DOI:** 10.1038/s41598-017-14415-8

**Published:** 2017-10-30

**Authors:** R. Charlotte Eccleston, Peter V. Coveney, Neil Dalchau

**Affiliations:** 1 Centre for Computational Science, Department of Chemistry, University College London, London, WC1H 0AJ UK; 2 CoMPLEX, University College London, London, WC1E 6BT UK; 3 Microsoft Research, Cambridge, CB1 2FB UK

## Abstract

The rate of progression of HIV infected individuals to AIDS is known to vary with the genotype of the host, and is linked to their allele of human leukocyte antigen (HLA) proteins, which present protein degradation products at the cell surface to circulating T-cells. HLA alleles are associated with Gag-specific T-cell responses that are protective against progression of the disease. While Pol is the most conserved HIV sequence, its association with immune control is not as strong. To gain a more thorough quantitative understanding of the factors that contribute to immunodominance, we have constructed a model of the recognition of HIV infection by the MHC class I pathway. Our model predicts surface presentation of HIV peptides over time, demonstrates the importance of viral protein kinetics, and provides evidence of the importance of Gag peptides in the long-term control of HIV infection. Furthermore, short-term dynamics are also predicted, with simulation of virion-derived peptides suggesting that efficient processing of Gag can lead to a 50% probability of presentation within 3 hours post-infection, as observed experimentally. In conjunction with epitope prediction algorithms, this modelling approach could be used to refine experimental targets for potential T-cell vaccines, both for HIV and other viruses.

## Introduction

The human immunodeficiency virus (HIV) is a fast mutating lentivirus that infects and eventually depletes T helper (Th) cells which are integral to adaptive immunity in vertebrates. As Th cell numbers decline, the infected individual becomes more susceptible to opportunistic infections and tumours. Therefore, HIV-infected individuals usually progress to acquired immunodeficiency syndrome (AIDS) within 10 years. However, 10–15% of people progress rapidly within three years of infection, whereas 5–10% remain asymptomatic for over 10 years^1^. People who control HIV for many years are known as long term non-progressors (LTNPs). Long-term non-progression is not linked to viral defects^2^, but is due to the response of the infected individual’s immune system. For example, an individual experiencing fast progression to AIDS, can transmit HIV to another individual who then becomes an elite controller (EC)^3^. Elite controllers comprise less than 1% of LTNPs, and have undetectable viral loads. More generally, understanding the factors that influence HIV progression rates and lead to long term control will aid in the design of vaccines, immunotherapy and personalised treatments.

Differing rates of progression in HIV-infected individuals are known to be linked to class I proteins of the Major Histocompatibility Complex (MHCI), which in humans are the human leukocyte antigen (HLA) proteins^4,5^. MHCI molecules present peptides arising from intracellular protein turnover at the surface of nucleated cells, allowing cytotoxic T-lymphocytes (CTL) the opportunity to detect intracellular pathogens such as viruses and bacteria, or cancerous mutations in self-proteins. Immunoproteasomes cleave peptides of lengths between 8 and 15 amino acids from proteins. Peptides are then transported into the endoplasmic reticulum (ER) via transporter associated with antigen processing (TAP) molecules, where they can bind to MHCI. Complexes formed may then be transported from the ER to the cell surface. If the peptide-MHCI complex has high stability (low off-rate), then the peptide will be displayed at the cell surface for a longer duration, serving to enhance the immunogenicity of the peptide^6^.

Several HLA alleles such as B*58, B*57, B*27 and B*44 have been found to be over-represented among LTNPs and ECs, and they are associated with Gag-specific CTL responses^7–11^. Many of these Gag-specific peptides originate from highly conserved regions of the Gag protein sequence^12^ and so escape mutations will likely lead to diminished viral fitness. For example, the known T242N escape mutation in the HLA-B*57/B*58:01 restricted Gag epitope TW10 (TSTLQEQIGW) leads to diminished viral replication capacity^5^, as does the A163G mutation of the similarly restricted Gag KF11 (KAFSPEVIPMF) epitope^13,14^. There is also an association among the subset of infected individuals who progress rapidly to AIDS and the expression of the alleles HLA-B*35 and -B*18. These fast-progressing alleles are associated with CTL responses against non-Gag epitopes, such as those from the Nef and Env proteins^15^. The Env and Nef proteins are both highly variable, with Env being the most variable sequence in the HIV genome^7^ and mutations in these epitopes are fitness neutral^16^. These observations would suggest that the strong association with Gag-specific T-cell responses and control of HIV progression is due to the Gag protein sequence being highly conserved in the HIV genome. However, Pol protein sequence is known to be the most conserved in the HIV genome^7^, but association between Pol-specific T-cell responses and immune control of HIV is not as convincing.

Another factor that might influence why Gag is so dominant in HIV control is peptide abundance. There are approximately 4900 copies of Gag protein per HIV virion^17^, and Gag is the most abundant HIV protein in the host cell cytoplasm during replication, with a Gag-Pol ratio in both the virion and during replication of 20:1^18^. There is a correlation between the abundance of epitopes from Gag proteins p17 and p24 in the endoplasmic reticulum (ER) and CTL immunodominance, but MHCI affinity only moderately influences the CTL response^19^. Since 99% of peptides in the cytoplasm degrade before encountering MHCI molecules in the ER^20^, peptides cleaved from high abundance proteins will have a greater probability of cell-surface presentation. Intracellular protein abundance is therefore an important factor when trying to predict CTL epitopes.

The development of a successful HIV T-cell vaccine would require the identification of dominant and sub-dominant T-cell epitopes originating from conserved regions of the HIV genome, to minimise the impact of escape mutations. T-cell epitopes can be discovered using high-throughput experimental methods, but such methods can only scan a limited number of proteins and MHCI alleles at a given time. Furthermore, such procedures are expensive and it is infeasible at present to perform full scans of all potential T-cell epitopes for complex viruses such as HIV^21^. The construction of models that predict the cell surface presentation of viral or cancerous peptides is therefore of paramount concern in biology and medicine. Towards the goal of predicting T-cell epitopes, there has been considerable work. Machine learning algorithms have been applied to large sets of experimental data, with artificial neural networks and matrix-based models trained to make reliable predictions about which sequences are likely to be immunogenic^22,23^. The Immune Epitope Data Base (IEDB) MHCI processing tool takes a protein amino acid sequence as input and predicts which peptide sequences will be produced. For each peptide the tool provides predictions of proteasomal cleavage, TAP transport and MHCI binding^24^ and provides a measure of how likely it is for a specific peptide sequence to be presented on the cell surface by the chosen MHCI allele.

In this paper, we present a new predictive mechanistic model of cell surface peptide presentation following HIV infection. The model combines the peptide specific properties predicted by the IEDB MHCI processing tool and a mechanistic model of infection that includes intracellular peptide abundance and viral lifecycle kinetics, factors that have previously not been considered in a quantitative framework^25^. The resulting model is a large system of ordinary differential equations (ODEs). The aim is to improve our understanding of the dominant factors within the intracellular peptide processing pathway and to be able to use such a model to accurately predict the timing and hierarchy of viral peptide presentation on the cell surface at different stages post infection of a cell. By combining viral protein kinetics with predictions of relative proteasomal cleavage rates we can model the dynamics of peptide production in the cytoplasm, which subsequently impacts the concentration of these peptides in the ER, and thus the amount of peptide available for binding to MHCI. In the context of viruses such as HIV, early forming proteins such as Rev, Tat and Nef, are translated several hours before the remaining HIV proteins, including the important structural proteins Gag, Pol and Env. Using the model, we assess the impact of viral protein kinetics on peptide presentation. We also compare the importance of factors such as protein synthesis, proteasomal cleavage and peptide-MHCI affinity on the peptide abundance at the cell surface, thereby gaining a greater understanding of the intracellular antigen processing pathway.

## Results

### IEDB predictions cannot explain immunodominance of Gag epitopes

**Figure 1:**
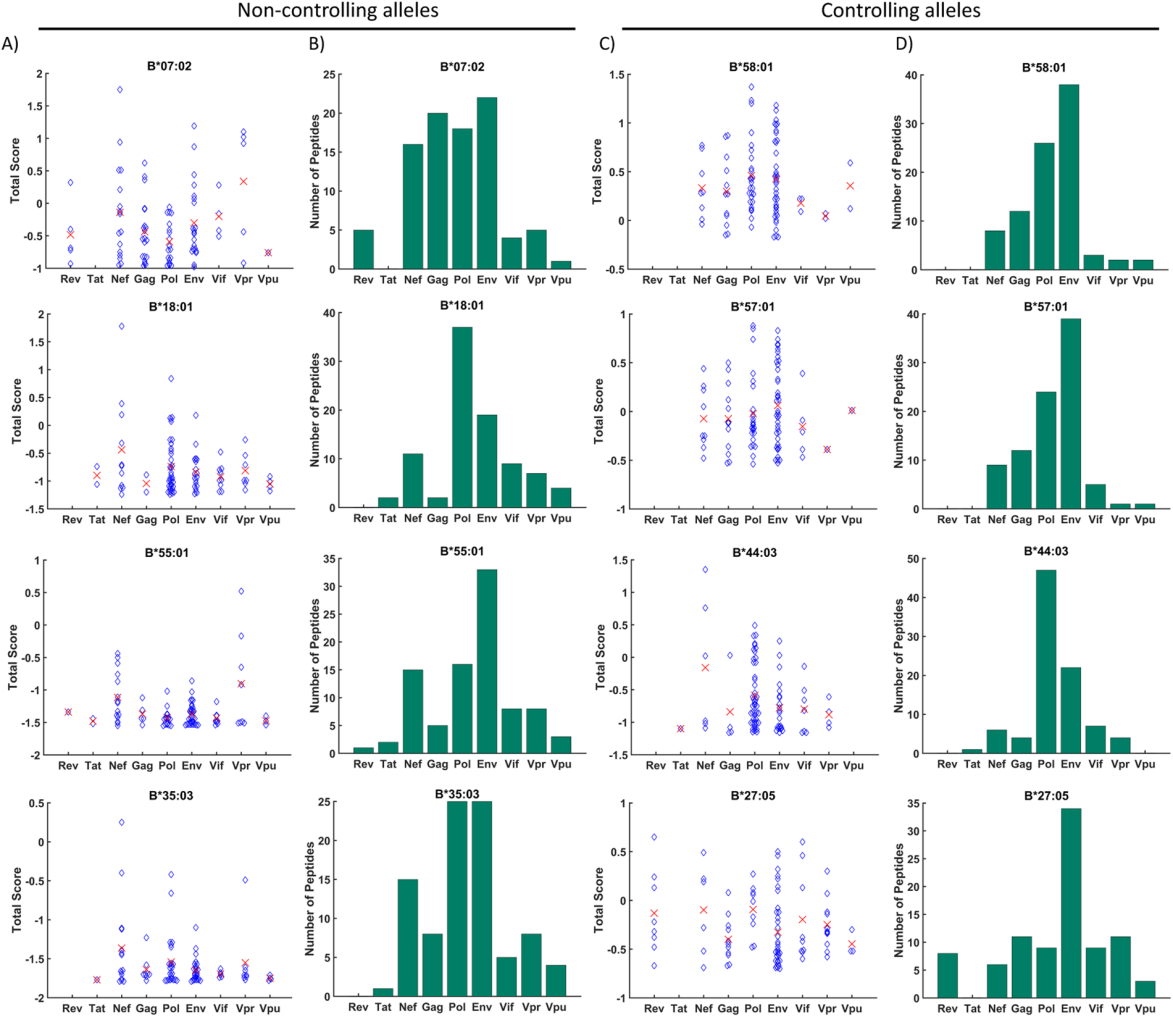
The IEDB prediction tool suggests that Pol and Env produce the majority of peptides presented on HLA molecules. The IEDB MHCI processing tools were used to analyse the distribution of the top 1% of HIV-1-derived peptides predicted to be presented on MHCI molecules. The predictions were made for controlling alleles, HLA-B*58:01, B*44:03, B*57:01 and B*27:05, and non-controlling alleles B*07:02, B*18:01, B*55:01 and B*35:03. (**A,C**) The IEDB Total Score for each peptide is plotted according to which protein they originate from. The red crosses indicate the average total score of peptides from each protein. (**B,D**) The number of peptides in the top 1% is compared for each protein.

To determine the extent to which the association between Gag epitopes and HIV control may be predicted from static models of peptide processing and MHCI binding, we used the MHCI processing tools from IEDB (see Methods). We used the HIV-1 clade C proteome as input to the tool, which predicts the peptidome of an amino acid sequence, the probability of proteasomal cleavage, TAP affinity and the affinity (IC_50_) between the peptide and chosen MHCI allele (see Methods for further details). The tool combines these three measures into a ‘Total Score’, which is designed to be proportional to cell surface abundance of the peptide. Thus, the higher the score, the more immunodominant the peptide. We selected and compared the peptides within the top 1% Total Score for four alleles associated with long term control of HIV: HLA-B*58:01, B*57:01, B*27:05 and B*44:03^7–11^, and four alleles associated with fast progression: HLA-B*18:01, B*35:03, B*07:02 and B*55:01^15,26^. Finally, we compared each HIV protein by taking the average Total Score of peptides from each protein (Fig. 1A,C), and recorded the total number of peptides from each protein that were predicted to be presented (Fig. 1B,D).

Due to the strong association with the presentation of Gag epitopes, we would expect that for the alleles associated with long term non-progression, the highest Total Scores would come from Gag peptides and on average Gag peptides would have a higher Total Score. However, the average Gag Total Score was one of the lowest for the controlling alleles, with the highest average scores coming from Pol, Env, Nef and Vif in general (Fig. 1C). Similarly, for the controlling alleles HLA-B*58:01, B*44:03 and B*57:01, Pol and Env were the two proteins predicted to produce the largest number of binding peptides to the controlling alleles (Fig. 1D), and not Gag.

When comparing progressors to LTNPs, we found that the average Total Score for the peptides presented by the non-controlling alleles B*35:03 and B*55:01 was much lower for each protein than the controlling alleles (Fig. 1A,B), which suggests that these alleles do not present immunogenic epitopes, and so are unable to control the spread of the virus. However, the non-controlling B*07:02 allele was predicted to bind a similar number of Pol, Env and Gag peptides. Furthermore, the B*18:01 allele was predicted to bind a similar number of peptides overall as the controlling alleles, and with a similar range of Total Scores. Focussing on Gag-derived peptides alone, we found that the highest average Total Score is associated with the controlling allele B*58:01, and the lowest average Total Score is associated with the non-controlling allele B*35:03. However, again we found no obvious distinction between the average Gag peptide Total Scores between the chosen set of controlling and non-controlling alleles that could explain their observed differences in rates of disease progression. In fact, from these predictions, we would expect Pol peptides to control HIV progression, as Pol is a highly conserved sequence and yields a large number of peptides with high Total Scores. In contrast, the Env sequence is highly variable^27^, so even though it also produces many peptides with high Total Scores, the higher probability of escape mutations reduces its immunogenicity.

Borghans *et al*.^28^ compared the predicted ranks of peptides from different HIV-1 proteins between a group of controlling alleles (HLA-B*27:05, -B*57:01 and -B*58:01) and a group of non-controlling alleles (HLA-B*35:03 and -B*53:01). When comparing the ranks of the top 3 best binding Gag peptides they found the controlling group had a significantly higher preference for Gag than the non-controlling group. They also found that the non-controlling group had a preference for Nef peptides compared to the controlling group when analysing the top 3 best Nef-derived binders. Significant differences in the preferences for Vpu, p17, Vif and Ref peptides were also observed but it was concluded that the median ranks of these peptides were so high that the differences were most likely not physiologically important.

**Figure 2:**
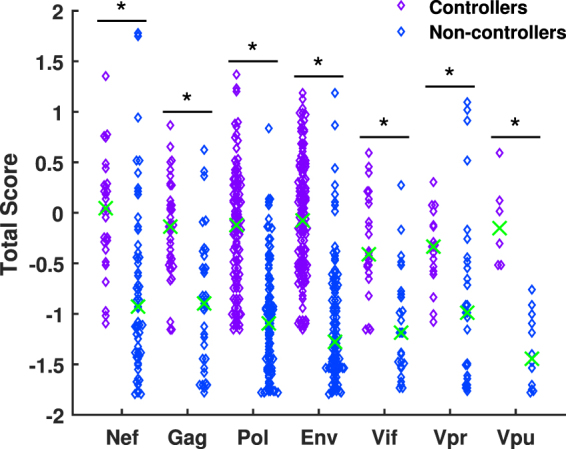
Controlling alleles process and present HIV peptides more efficiently than non-controlling alleles. The IEDB processing tool Total Scores calculated in Fig. 1 were combined into sets of HIV peptides for controlling and non-controlling alleles. Their median (green crosses) were compared using a Wilcoxon rank-sum test. A significantly higher median Total Score when binding to the controlling group was observed for all HIV proteins included in the analysis, suggesting controlling alleles preferentially bind HIV peptides in general compared to non-controlling alleles.

We carried out a similar analysis by comparing the median predicted Total Score of all peptides in the top 1 % of each allele when grouping the alleles by their association with long-term control, and then asking whether the Total Scores are differ significantly, using the Wilcoxon rank-sum test (as used by Borghans *et al*.^28^; Fig. 2). A statistically significant difference was observed for all HIV proteins considered, suggesting that controlling alleles preferentially bind HIV peptides from Nef (*p* = 3.6 × 10^−6^), Gag (*p* = 2.4 × 10^−6^), Pol (*p* = 3.1 × 10^−18^), Env (*p* = 1.5 × 10^−24^), Vif (*p* = 1.2 × 10^−5^), Vpr (*p* = 0.0058) and Vpu (*p* = 1.0 × 10^−4^) in general compared to non-controlling alleles (Ref and Tat were not included due to the low numbers of peptides from these proteins in the top 1 %). This analysis suggests that controlling alleles are better suited in general to present peptides from the entire HIV genome although we could conclude that we would expect the two proteins with the lowest p-values, Pol and Env to be associated with control of HIV. However, as noted above, this IEDB-based analysis does not provide any explanation for the immunodominance of Gag epitopes.

One conflating factor in using the IEDB predictors to establish whether a given HLA allele is likely to control HIV infection is that they do not account for differential intracellular abundance and kinetics of the proteins from which the peptides are cleaved. Therefore, to determine whether the kinetics and abundance of HIV proteins can be determinants of HIV control, we must consider dynamic models of viral infection and MHCI presentation.

### Construction of a combined model of HIV infection and peptide-MHCI presentation

**Figure 3:**
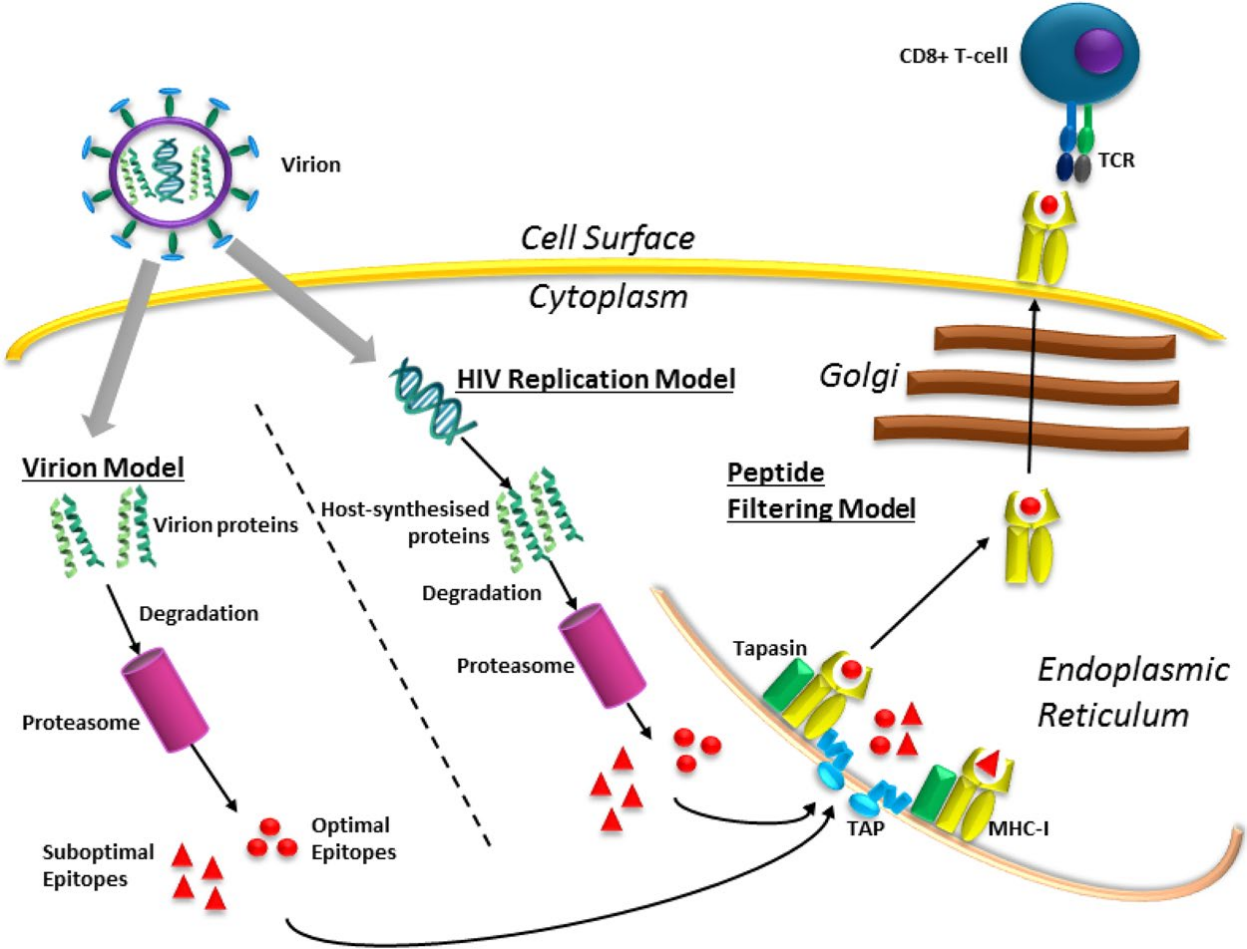
Combined model of HIV-1 infection and cell surface peptide presentation on MHCI molecules. Diagrammatic representation of the combination of the separate models that comprise the combined model: the HIV kinetics models^18,29,30^, and the peptide filtering model^33^.

To address the question of HLA-dependent control of HIV from a dynamical perspective, and simultaneously gain greater understanding of the mechanisms underlying antigen presentation, we constructed a combined model of HIV intracellular kinetics and MHCI peptide presentation (Fig. 3). Our model accounts for the impact viral protein dynamics have on peptide presentation, whilst using the predictive power of the IEDB processing tool to provide relative values for peptide specific parameters. We attempted to include as many of the known sequence-dependent steps of the intracellular antigen presentation pathway, to enable us to compare which are most influential, how presentation differs between early and late appearing proteins, and between high abundance and low abundance proteins. The model constructed enables us to produce time-dependent predictions of the cell surface peptidome associated with specific HLA alleles following HIV infection and viral genome integration, comparing these predictions between controlling and non-controlling alleles.

The model incorporates three existing ODE (ordinary differential equation) models of HIV-1 intracellular kinetics: Kim & Yin^29^, Reddy & Yin^18^ and Wang & LuHua^30^. HIV has been studied in detail and all three of these models are based on a wealth of experimental data that characterizes the rates of HIV mRNA transcription, ratios of HIV protein translation and half-lives, protein cytoplasmic concentrations and interactions with host proteins, and the mechanisms and kinetics of viral replication and virion budding. The articles introducing each of the three models focussed on a different aspect of HIV intracellular kinetics. The combined model is a large system of coupled ODEs which uses experimentally determined reaction rates, or rates inferred from the IEDB processing tool, to describe the dynamic presentation of HIV-1 peptides following the infection of a cell by a HIV-1 virion. The model describes the steps of HIV-1 intracellular kinetics, including in the translation of the HIV-1 proteasome, beginning with the synthesis of full-length HIV-1 mRNA following the integration of an HIV-1 genome in to the host genome. The full-length mRNA encodes for the proteins Gag and GagPol and is spliced in the nucleus in to singly-spliced mRNA, which encodes for the proteins Env, Vif, Vpr and Vpu. The singly-spliced mRNA is spliced to form multiply-spliced mRNA which encodes for the regulatory proteins Rev and Tat. Only multiply-spliced mRNA can independently export to the cytoplasm, where Rev and Tat are translated and then re-enter the nucleus so Rev can bind to the larger viral mRNAs and allow for nuclear export, and so Tat can increase the rate of transcription of full-length mRNA. By combining the three models we are able to model the intracellular kinetics of the HIV-1 proteins Gag, Pol, Env, Rev, Tat and Vif, including budding of new virions. Inside each virion there are approximately 4900 copies of Gag and 700 copies of Vpr, a ratio of 7:1^17^. The Pol protein is found at a ratio 1:20 compared to Gag^31^, with around 245 copies per virion. The copy numbers for the other proteins that comprise the HIV virion are as follows: Vif, 101^30^; Env, 282^18^; Nef, 150^32^; Vpu, unknown; Tat, none; Rev: none. We therefore set the synthesis rates of the proteins and degradation rates not included in either of the models, Vpr, Vpu and Nef, so that their numbers in the virions matched the measured values, where available, using experimentally measured half-lives (see Table S1 for parameter values).

**Figure 4:**
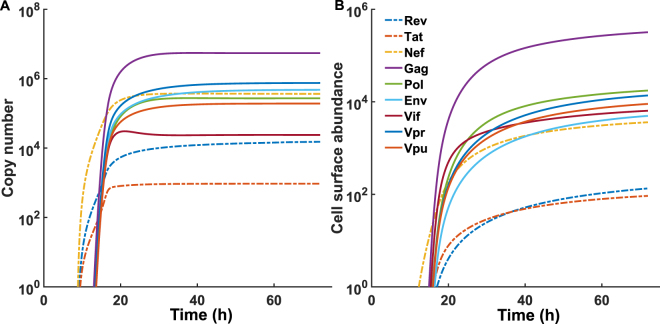
Example simulation of the combined model of HIV infection and peptide-MHCI presentation. (**A**) Simulated levels of HIV-1 proteins produced during replication (calculated deterministically). The complete model that produces all HIV-1 proteins is a combination of three existing models Kim & Yin^29,52^, Reddy & Yin^18^, and Wang & LuHua^30^. (**B**) Simulated cell surface abundance of *efficient* peptides derived from each protein, considered to have *u*_*i*_ = 10^−5^ s ^−1^, a proteasomal cleavage *pc*_*i,j*_ = 0.1, and a fast supply rate ${g}_{i}=0.08$ peptides s^−1^ to the ER.

The combined model of HIV kinetics enabled us to simulate the dynamics of all nine HIV-1 proteins following the infection of a single cell by a virion particle (Fig. 4A). We then combined this model with the peptide filtering model^33^, which describes MHCI peptide binding and presentation. In the peptide filtering model, a peptide is supplied to the ER where is it available for binding to MHCI or tapasin-MHCI complexes. The presence of tapasin increases the unbinding rate of the peptide from the MHCI molecule, and so acts as a filtering mechanism by which only those peptides with a high affinity for the MHC in question stay bound long enough to egress to the cell surface. Therefore, cell surface abundance of peptide-MHCI complexes ([*MP*]_*i*__cs_; here *i* denotes a specific peptide sequence) is approximately inversely proportional to the square of its unbinding rate *u*_*i*_ (see Supplementary Table S4). To combine the model of HIV intracellular kinetics and the model of peptide filtering we included the steps of peptide cleavage during protein degradation within the proteasome, peptide degradation in the cytoplasm, and peptide supply to the ER via TAP, according to

1 $$\frac{d{[{P}_{i}]}_{{\rm{cyt}}}}{dt}=p{c}_{i,j}\cdot {k}_{j}[{{\rm{Prot}}}_{j}]-{g}_{i}{[{P}_{i}]}_{{\rm{cyt}}}-{d}_{{P}_{i,C}}{[{P}_{i}]}_{{\rm{cyt}}}$$

where *P*_*i*_ is the peptide of sequence *i*, and its cytoplasmic/ER concentration is given by [*P*_*i*_]_cyt_ and [*P*_*i*_] respectively. The peptide *P*_*i*_ is cleaved from protein *Prot*_*j*_ with rate constant and $p{c}_{i,j}\cdot {k}_{deg}^{Pro{t}_{j}}$, which is the probability that the peptide will be produced via the degradation of one protein of type $j$. Peptide *P*_*i*_ can then be degraded in the cytoplasm with rate constant ${d}_{{P}_{i,C}}$ or transported to the ER with rate constant *g*_*i*_. The term ${g}_{i}{[{P}_{i}]}_{{\rm{cyt}}}$ acts as the supply rate of peptide *P*_*i*_ to the ER and so connects the model of HIV protein kinetics with the model of peptide-MHCI binding (see Methods section for detailed description of model and parameter values). We use the IEDB peptide MHC-I processing tool (http://tools.iedb.org/processing/) to predict which peptide sequences will be produced by each HIV-1 protein, using the amino acid sequence of the HIV-1 clade C proteome as input to the tool. The processing tool provides predictions of the peptide-MHC affinity and the proteasomal cleavage probability that can be used to parameterise Equation 1.

### Simulation of HIV-1 epitope presentation: Gag peptides dominate at the cell surface

Initially, we considered the HIV-1 replication kinetics with deterministic rate equations as the proteins are synthesised to high abundance following reverse transcription. In keeping with the existing models of HIV kinetics, the Gag protein had the highest cytoplasmic and virion abundance. Experimental evidence suggests that the Gag:Pol ratio in the virion of 20:1 is maintained in the cytoplasm^31^. Initially, we considered the presentation of an *efficient* peptide for each HIV protein, to determine which will produce the most abundant peptides on the cell surface. The efficient peptide was defined as having a high affinity for MHCI encoded as a low unbinding rate (*u* = 1 × 10^−5^ s^−1^), a high probability of proteasome cleavage ($p{c}_{i,j}=0.1$), and a rate of supply into the ER (${g}_{i}=0.08$ peptides s^−1^). In this way we are initially not considering any specific peptides but looking at peptide presentation as determined only by the differences in the kinetics of the originating protein.

The early synthesised regulatory proteins, Rev, Tat and Nef, are the first to appear in the cell cytoplasm at around 9 hours post infection, and are the first HIV proteins translated before the downregulation of MHC-I by Nef. Peptides derived from these regulatory proteins are frequently targets of CTL response, and so may be good targets for a HIV-1 vaccines^34^. Rev and Tat shuttle between the cytoplasm and the nucleus, in order to regulate HIV mRNA nuclear export, and viral genome translation respectively^29^. The model predicts that this rapid shuttling means that the Rev and Tat proteins accumulate slower in the cytoplasm that Nef, and even at steady state the cytoplasmic abundance of these two early proteins is much lower than that of all other HIV-1 proteins (Fig. 4A). As a consequence, out of the three early HIV proteins, the model predicts that only an optimal epitope deriving from the Nef protein will be presented significantly early, around 12 hours post-infection, whilst the optimal epitopes from Rev and Tat do not appear on the cell surface in substantial amounts until after the optimal epitopes from the later proteins, Gag, Pol, Env, Vif, Vpr and Vpu, which appear at around 15–16 hours post infection (Fig. 4B). This suggests that any benefit that would be gained by targeting epitopes from the early HIV proteins before MHC-I downregulation would only be applicable in the case of Nef epitopes and not Rev or Tat.

As would be expected, the Gag peptide dominates cell surface abundance, with a Gag:Pol ratio of 18:1, a Gag:Vpr ratio of 23:1 and a Gag:Env ratio of 64:1. While there are very few published measurements of the cell surface abundance of HIV peptides, it has been determined that the ratio in the presentation of two HLA-A2 restricted peptides, gag 77–85 (SLYNTVATL) and pol RT 476–484 (ILKEPVHGV), is around 30:1^35^. The authors further suggest that this is consistent with the ratios of Gag and Pol in the virion and cytoplasm, around 20:1. Whilst we would expect the Gag epitope to be the most abundant on the cell surface due to the high Gag copy number, the ranking of the cell surface abundance for the epitopes deriving from the other proteins does not necessarily follow the ranking of their cytoplasmic abundances. The Vpr protein is the second most abundant in the cytoplasm, but the Pol peptide is the second most abundant on the cell surface. Similarly, the Env protein is the third most abundant in the cytoplasm, however, its epitope is only the sixth most abundant on the cell surface. This highlights how important the trade-off between protein synthesis and degradation is for peptide cell surface presentation: Vpr is a very stable protein^36^, and so whilst it is in high abundance in the cytoplasm, it degrades slowly, producing few peptides per unit time. A similar argument can be used to explain the discrepancies in the ranking of the Env protein and its epitope. Therefore, when trying to predict whether an epitope would be presented on the cell surface it is important to not only consider the abundance of the protein from which it originates but also the rate at which it degrades.

### Differential sensitivity analysis of the combined model

**Figure 5:**
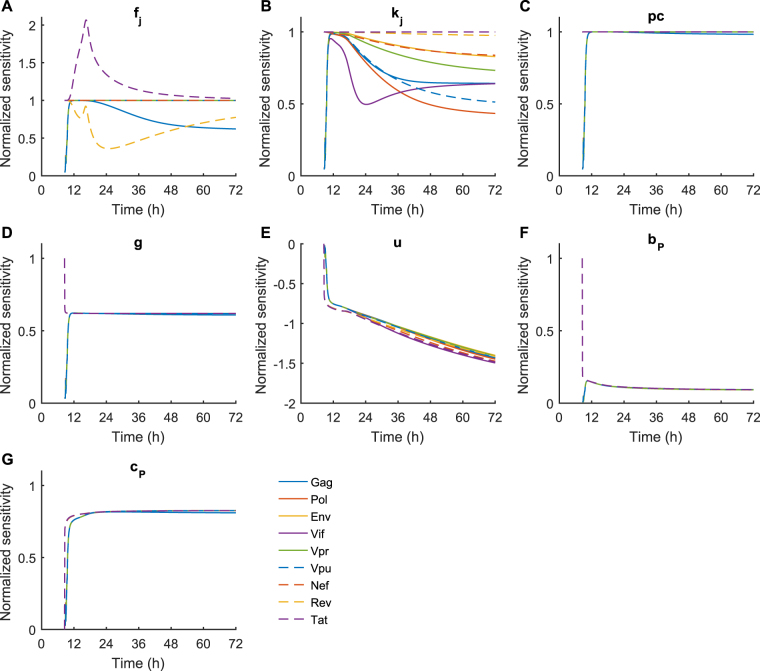
Sensitivity analysis of the combined model. We calculated the sensitivity of the cell surface presentation of optimal epitopes (as in Fig. 4) to seven of the model parameters: probability of protein translation *f*_*i*_ (*j* denotes the protein), cytoplasmic degradation *k*_*j*_, proteasomal cleavage probability *pc*, supply rate to the ER *g*, peptide-MHCI unbinding rate *u*, peptide-MHCI binding rate *b*, and the peptide-MHC-tapasin binding rate *c*_*P*_. The sensitivities were calculated using the CVODES module of the SUNDIALs package^63^, then normalised, as described in the Methods section.

To establish a more comprehensive insight into the dependency of the model on the underlying parameters, we performed a differential sensitivity analysis on the cell surface abundance, [*MP*_*i*_]_cs_, of an epitope for seven of the nine HIV proteins separately (Fig. 5; see Methods for details). To enable comparison of several parameters of the model, we computed the normalised sensitivity coefficients, which approximate report the scaling of the model behaviour to the parameter of interest^37^. In particular, a normalised sensitivity coefficient of 1 indicates a positive linear dependence on the parameter, while a coefficient of −2 indicates an inversely quadratic dependence. Applied to the combined model, there was a mostly linear dependency upon protein synthesis *f*_*j*_ for most proteins as time progressed (Fig. 5A). Presentation of Tat and Rev peptides had transiently nonlinear dependencies on their protein synthesis rates, which might have resulted from the effects of nuclear transport of these proteins. A more significant difference was for presentation of Gag peptides, which are associated with much higher protein synthesis rates than the other HIV proteins (Fig. 5A). Gag presentation showed a sublinear dependence on protein synthesis, which may be caused by the rapid accumulation of Gag epitopes resulting in being limited instead by ER translocation of cytoplasmic peptide. Interestingly, the dependence of [*MP*_*i*_]_cs_ on protein degradation (*k*_*j*_) was sublinear as time progressed for each protein considered (Fig. 5B), meaning cell surface abundance is in general less sensitive to changes in the protein degradation rate than the synthesis rate. Finally, we observed an almost constant linear dependence on proteasomal cleavage ($pc$; Fig. 5C), but a sublinear dependence on TAP-mediated translocation ($g$; Fig. 5D). It is possible that the low influence of TAP could be due to its co-evolved specificity with the proteasome^38^.

#### Cell surface presentation is dominated by peptide processing initially, then gives way to peptide off-rate

With regards to the peptide-dependent parameters, there was a consistently linear dependence of peptide-MHCI cell surface abundance on proteasomal cleavage probability *pc* for each protein (Fig. 5C), whereas the dependence on the peptide ER supply rate *g* was sublinear (Fig. 5D), suggesting proteasomal cleavage is more important to an epitopes immunogenicity than the ER supply rate, and thus the epitopes affinity to TAP. The normalised sensitivity with respect to small increases in the value of the peptide-MHC unbinding rate *u* became more negative with time, and approached −1.5 by 72 hours post infection (Fig. 5E), and eventually plateaus at around −2 in equilibrium (data not shown). This near-quadratic dependency agrees with the relation ${[M{P}_{i}]}_{{\rm{cs}}}\propto \mathrm{1/}{u}_{i}^{2}$ proposed previously^33^.

The normalised sensitivity with respect to the rates of peptide binding free MHCI, *b*_*P*_ (Fig. 5F), and tapasin-bound MHCI, ${c}_{P}$ (Fig. 5G), were both sublinear, but small increases in ${b}_{P}$ result in a smaller increase in cell surface abundance than small increases in ${c}_{P}$ do. In the peptide filtering model^33^, ${c}_{P} > {b}_{P}$, and $[MT]\gg [M]$, and therefore the peptide binding rate to tapasin-bound MHCI is more important to overall cell surface peptide abundance. Overall, this sensitivity analysis suggests that if T-cell immunodominance hierarchies are established early following infection, then the steps of peptide processing, such as protein synthesis and proteasomal cleavage, as opposed to MHCI stability alone, could be more important than previously thought. As time progresses, however, the MHC stability becomes more important, as demonstrated by the increasing normalised sensitivity of ${[M{P}_{i}]}_{{\rm{cs}}}$ with respect to the peptide-MHCI unbinding rate ${u}_{i}$.

### Simulated HIV-1 peptide presentation by controlling and non-controlling HLA alleles

**Figure 6:**
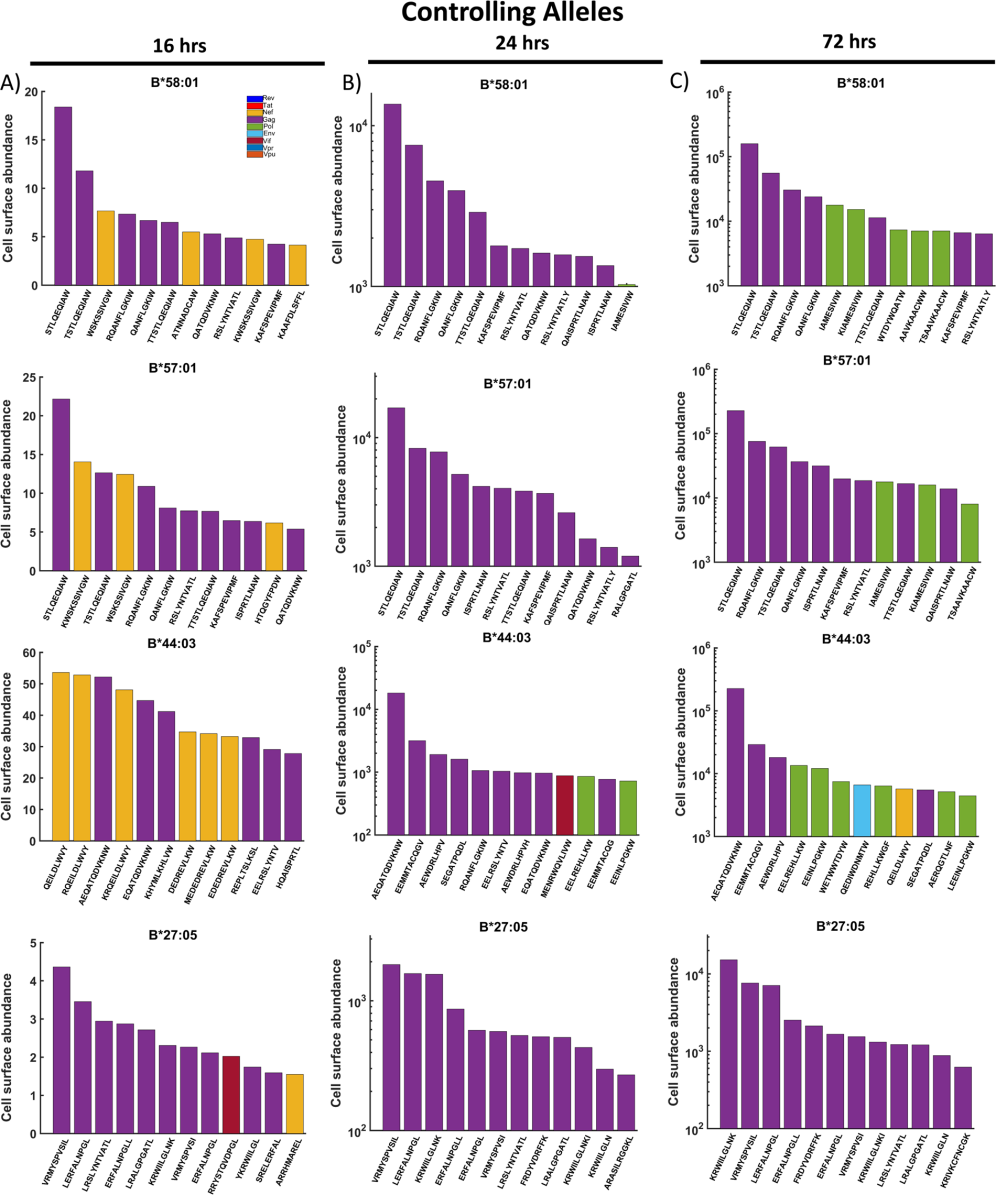
Controlling alleles all demonstrate sustained Gag peptide presentation, and/or combined Gag and Pol peptide presentation at later times post infection. The combined model was used to predict the cell surface abundance of HIV-1 peptides in controlling alleles (B*58:01, B*57:01, B*44:03 and B*27:05) over time. The top 12 most abundant peptides at (**A**) 16, (**B**) 24 and (**C**) 72 hours post-infection are shown, with bar colours indicating the originating protein. All controlling alleles presented several Gag peptides by 16 hours, with the number of Gag peptides increasing by 24 hours post-infection. The presentation of Gag peptides at high abundance is sustained up to 72 hours post-infection.

**Figure 7:**
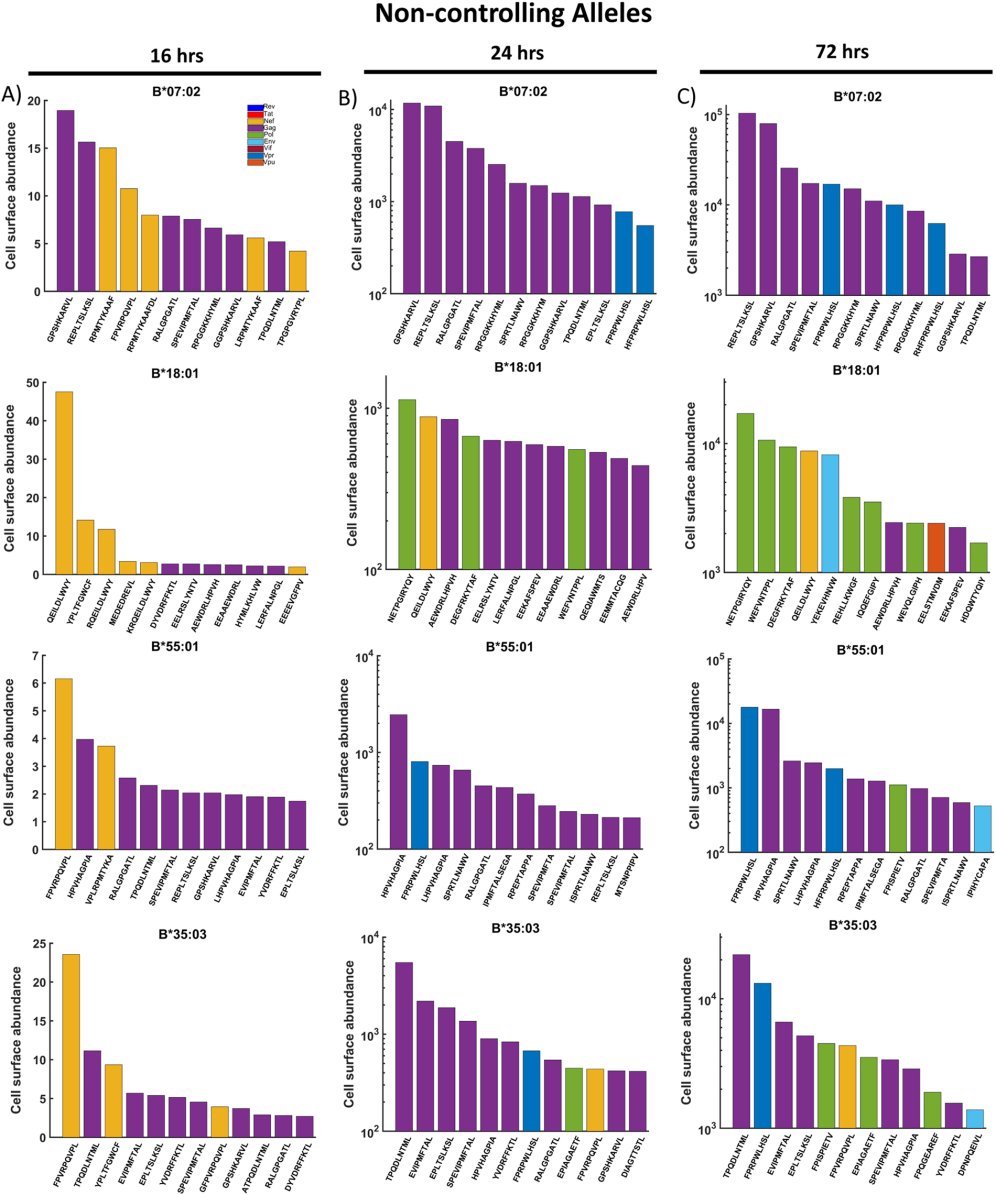
Non controlling alleles are either unable to sustain high levels of Gag peptide presentation, or present a combination of Gag and Vpr peptides at later times post infection. The combined model was used to predict the cell surface abundance of HIV-1 peptides non-controlling alleles (B*07:02, B*18:01, B*55:01 and B*35:03) over time. The top 12 most abundant peptides at (**A**) 16, (**B**) 24 and (**C**) 72 hours post-infection are shown, with bar colours indicating the originating protein.

We performed simulations of peptide-MHCI presentation of HIV-derived peptides for up to 72 hours post infection using the combined model, and compared four controlling alleles HLA-B*58:01, B*57:01, B*27:05 and B*44:03^7–11^ and four non-controlling alleles, HLA-B*18:01, B*35:03, B*07:02 and B*55:01^15,26^. We then analysed the twelve most abundant peptides presented on the cell surface at 16, 24 and 72 hours post infection, and determined which protein they originated from (Figs 6 and 7).

#### Most alleles transiently present Nef-derived peptides but become dominated by Gag-derived peptides

At 16 hours post-infection, the model predicts that all controlling and non-controlling alleles present a mixture of Nef (yellow bars) and Gag peptides (purple bars) (Figs 6A and 7A), with the exception of HLA-B*27:05, which also presents a single Vif peptide. Whereas, at later time-points, the Nef-derived peptides mostly become displaced, effectively limiting the impact of CTL responses mounted against these peptides. By 24 hours post-infection, the controlling alleles almost exclusively present Gag-derived peptides in the top 12, except for B*44:03, which presents 2 Pol and 1 Vif peptide (Fig. 6B). The non-controlling alleles present slightly more of a mixture. B*07:02 and B*55:01 present Gag and Vpr peptides, B*18:01 a mixture of Gag, Pol and Nef peptides, whilst B*35:03 presents Gag, Pol, Nef and Vpr peptides (Fig. 7B). By 72 hours post-infection, the controlling allele B*27:05 continues to present exclusively Gag peptides in the top 12, while the number of Pol peptides presented by B*58:01, B*57:01 and B*44:03 increases considerably (Fig. 6C). The non-controlling alleles continue to present more broadly across the HIV proteins (Fig. 7C). Of particular note is B*18:01, which presents mostly Pol peptides, and only 2 Gag in the top 12.

In summary, the distribution of peptides presented by controlling and non-controlling alleles is similar at 16 hours post-infection, however some distinctions arise at later time points. The top three most abundant peptides at 72 hours post-infection presented by all the controlling alleles are exclusively Gag peptides, though this is also true of the non-controlling B*07:02 allele. However, B*55:01 presents 1 Vpr and 2 Gag in the top three at both 24 hours and 72 hours, whilst B*35:03, which presents exclusively Gag in the top 3 at 24 hours post-infection, also presents 1 Vpr and 2 Gag in the top 3 by 72 hours post-infection. While Vpr peptides are present in the top 12 of three out of the four non-controlling alleles’ top 12 peptides (B*07:02, B*55:01 and B*35:03) by 72 hours post-infection, Vpr peptides were absent in the top 12 of all controlling alleles.

#### Non-controlling alleles present Vpr peptides in high average abundance

To provide a broader perspective of the appearance of peptides from specific proteins being presented, we analysed the *average* abundance of the peptides from each protein at 16, 24 and 72 hours post-infection (Supplementary Figures S1 and S2). At both 24 hours and 72 hours post-infection, B*58:01, B*57:01 and B*27:05 all presented Gag peptides with the highest average abundance, whilst for B*44:03, peptides from both Gag and Nef similarly high (Supplementary Figure S1B,C). Peptides from Nef, Pol and Env were also presented with high average abundance by B*58:01 and B*57:01 at 24 hours post-infection. Notably, however, the average abundance of Vpr peptides presented by B*58:01, B*57:01 and B*44:03 was very low. Whereas, from 24 hours post-infection, Vpr peptides had the highest average abundance for the non-controlling alleles B*07:02 and B*55:01, and the third highest abundance for B*35:03 (Supplementary Figure 2B,C). Therefore, when considering average peptide abundance and the distribution of the top 12 peptides, our simulations suggest that presenting a combination of Gag and Pol peptides provides better protection against HIV disease progression than presenting a combination of Gag and Vpr peptides. Whilst the non-controlling allele HLA-B*18:01 *does* present Pol peptides alongside Gag peptides, it is the Pol peptides that dominate the top 3, suggesting that control of HIV progression requires that the Gag peptides dominate and are complemented by Pol peptide presentation. Furthermore, when considering the average peptide abundances, this allele presents Nef peptides with highest average abundance at all time points, whereas for all controlling alleles, Gag is in highest average abundance by 72 hours post-infection.

**Figure 8:**
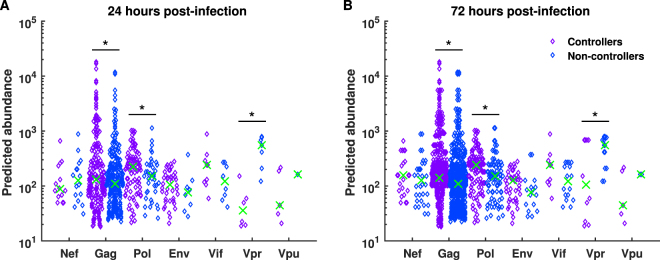
Controlling alleles prefer to present Gag and Pol peptides. The predicted abundance of the top 1% of HIV epitopes from different HIV proteins were grouped by controlling and non-controlling alleles. The median abundance of the top 1% predicted HIV peptides at (**A**) 24 hours and (**B**) 72 hours post infection was then compared using a Wilcoxon rank-sum test. At both time points, a significantly higher median abundance was observed for Gag and Pol peptides in the controller group, while a significantly higher median abundance was observed for Vpr peptides in the non-controlling group.

To assess how significant the differences predicted by our model are between the controlling and non-controlling alleles, we carried out a similar statistical analysis to before (Fig. 2). This time, we considered the top 1 % most abundant peptides from each allele and then grouped into controlling and non-controlling alleles. The median predicted cell surface abundance of peptides from each HIV protein was then analysed using a Wilcoxon rank-sum test. This was done for both 24 (Fig. 8a) and 72 (Fig. 8b) hours post infection, but not 16 hours post infection due to the low number of peptides actually presented. We found that at both time points, controlling alleles presented peptides from Gag (24 hrs: *p* = 0.0432, 72 hrs: $p=3.67\times {10}^{-7}$) and Pol (24 hrs: $p=0.0435$, 72 hrs: $p=0.0135$) with statistically significant higher abundance, whilst non-controlling alleles were shown to present Vpr (24 hrs: $p=0.0023$, 72 hrs: $p=0.0288$) peptides with statistically significant higher abundance. It is interesting to note that at 24 hours post infection the most significant difference between the controlling and non-controlling alleles is in the abundance of Vpr peptides, whilst at 72 hours post infection Vpr has the lowest significance, with the highest being associated with the abundance of Gag epitopes. This corroborates our findings from considering the top 12 most abundant peptides from each allele, that non-controlling alleles have a preference for presenting Vpr peptides in high abundance, whereas controlling alleles preferentially present Pol and Gag. This also supports our assertion that whilst machine learning algorithms such as IEDB can predict differences in MHC binding affinities and proteasomal cleavage probabilities, using these tools alone it is not possible to predict differences in presentation as the protein dynamics are not taken in to account.

#### The dynamic model predicts the presentation of known HIV-1 epitopes by controlling alleles

When comparing the outputs of the combined dynamic model (Fig. 6D) with those resulting from the static predictions of the IEDB processing tool (Fig. 1), we found that the known epitopes rank higher in our combined model. The model predicted that the controlling alleles would all present known HIV Gag epitopes associated with HIV control by 16 hours post-infection, and this presentation is sustained up to 72 hours post-infection (Fig. 6; Supplementary Table S2). For example, TW10 (TSTLQEQIAW) is a known p24 Gag epitope of B*58:01 and B*57:01^39^. The model predicts that TW10 is the second most abundant peptide presented by B*58:01, and alternates between the second and third most abundant peptide presented by B*57:01 (Fig. 6). However, the IEDB processing tool predicts TW10 to have only the 28^th^ highest Total Score for peptides binding to B*58:01 and only the 34^th^ highest score for B*57:01.

The known Gag p24 KF11 (KAFSPEVIPMF) epitope of B*58:01 and B*57:01^40^ is predicted to be the 11^th^ most abundant B*58:01 peptide at 16 hours post-infection, then increases to the 5^th^ most abundant peptide, before slipping down to 11^th^ place by 72 hours post-infection (Fig. 6). Similarly, KF11 increases its rank among the peptides presented on B*57:01, reaching 6^th^ position by 72 hours post-infection. However, KF11 is only ranked 23^rd^ and 16^th^ by the IEDB Total Scores for B*58:01 and B*57:01 respectively.

Furthermore, the known B*58 and B*57 restricted Gag epitope ISPRTLNAW (IW9)^28^ is the 11^th^ most abundant B*58:01 peptide at 24 hours post-infection, but then displaced by Pol peptides at 72 hours post-infection. IW9 is consistently presented by B*57:01 at all three time points, being the 10^th^ most abundant at 16 hours post infection, before increasing to 5^th^ place and remaining there by 72 hours. IW9 has the 88^th^ highest IEDB Total score for B*58:01 and the 54^th^ highest total score for B*57:01.

Finally, the known B*27:05 restricted Gag epitope KK10 (KRWIILGLNK)^41^ is the 6^th^ most abundant peptide at 16 hours post-infection and the most abundant by 72 hours post-infection (Fig. 6, bottom row), however it is only ranked 29^th^ by the IEDB Total Score. Also, known B*44:03 restricted epitope Gag AW11 (AEQATQDVKNW)^42^ is the third most abundant peptide by 16 hours post-infection, but then reaches and remains the most abundant peptide from 24 hours onwards, however it has only the 13^th^ highest predicted IEDB Total Score.

#### Peptide unbinding rates are more predictive of long-term cell surface presentation

Our simulations therefore clearly demonstrate that a peptides cell surface abundance may change significantly over time, and therefore become more or less immunogenic. For example, IW9 was only present in the top 12 most abundant peptides of B*58:01 transiently at 24 hours post infection but had been largely displaced by Pol peptides by 72 hours post infection. Our analysis suggests that this is due to the changing importance of peptide-specific parameters over time, as demonstrated by differential sensitivity analysis (Fig. 5). The Pol peptides (especially IAMESIVIW and KIAMESIVIW) that are predicted to be within the top 12 of both B*58:01 and B*57:01 at 72 hours post-infection both have very high predicted affinities for these alleles, and therefore have a low unbinding rate in our simulations. As is demonstrated in Fig. 5E, the peptide-MHC unbinding rate becomes increasingly important over time, and by 72 hours post-infection, is more influential to cell surface abundance than any other parameter, including protein synthesis and degradation. Therefore, these Pol peptides begin to appear because their very low unbinding rate compensates for the lower abundance of the Pol protein in the cytoplasm compared to Gag. IAMESIVIW Pol is a known B57/B58 restricted HIV epitope, and is predicted by IEDB to have the highest and second highest total scores of any HIV peptide for B*58:01 and B*57:01 respectively. However, it does not appear in the top presented peptides until later on in infection. Therefore, as we have demonstrated, assigning a static immunogenicity score ignores the dynamics occurring during viral replication and peptide presentation, as well as the changing importance of peptide specific parameters, which when combined, creates a very complex system that cannot be efficiently described by a single metric.

### Simulation of Virion-derived peptides reveals how Gag epitopes are likely to be presented within 3 hours of infection

The previous sections were concerned with monitoring cell surface peptide-MHCI presentation where the peptide originated from *de novo* protein synthesis following reverse transcription of the viral genome into the host cell. Given the time lag induced by reverse transcription, protein synthesis and then protein degradation, the first peptides do not arrive at the cell surface until approximately 10 hours post infection. However, the two protective Gag epitopes KF11 and KK10, restricted by HLA-B*57:01 and B*27:05 respectively, and the Pol KY9 B*27:05 restricted peptide have been detected on the surface of HIV-infected cells within 3 hours post-infection^43^, so could not have originated from *de novo* protein synthesis. Rather, these peptides presumably originated from the proteins that comprise the infecting virion(s). Therefore, we sought to determine whether our model could be used to simulate virion-derived peptide presentation. For peptides originating from *de novo* protein synthesis, the protein and peptide numbers become large ($\gtrsim 100$) within our time window of interest, justifying the use of deterministic ODE simulations. However, virion-derived peptides will be in much lower abundance, preventing use of deterministic simulations for analysis. Therefore, we specified a stochastic version of our combined HIV kinetics model as a system of chemical reactions (see Methods for details).

**Figure 9:**
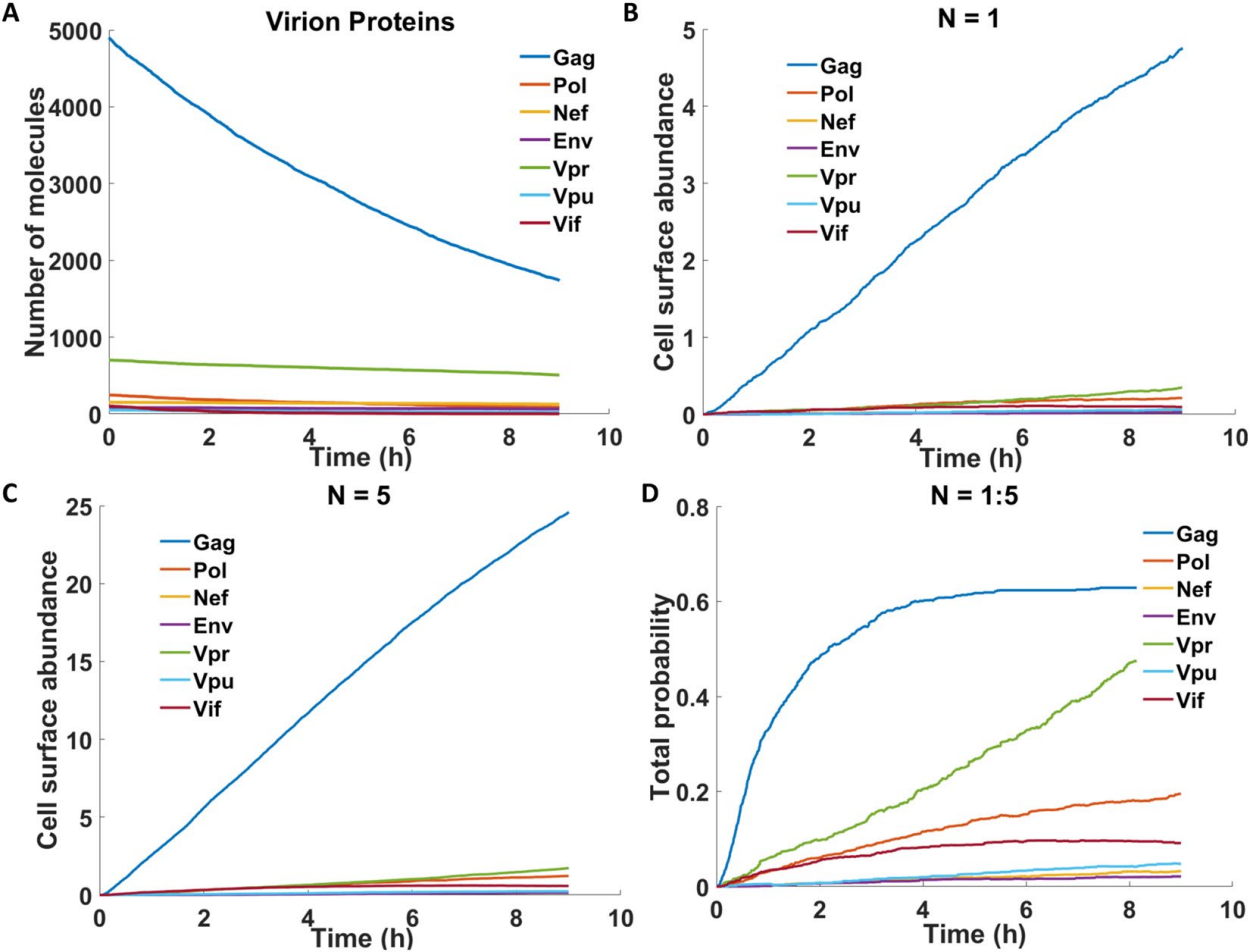
Stochastic simulation of virion-derived peptide presentation. The HIV virion model was simulated stochastically 300 times. (**A**) The mean HIV virion protein kinetics are shown for simulations of a single virion. (**B**) Mean cell surface abundance of optimal peptides from each of the 7 HIV proteins contained within a single virion. (**C**) Mean cell surface abundance of optimal peptides from 5 virions. (**D**) Using calculations of mean cell surface abundance, we approximated the probability of optimal peptide presentation using conditional probability, and by considering that the number of infecting virions is Poisson distributed with mean 1 (see Methods for details).

Because stochastic simulations are more computationally expensive than deterministic simulations, we simulated one *efficient* peptide for each virion protein (as opposed to multiple peptides per protein), similar to what was done in Fig. 4B. As before, the efficient peptide has a low unbinding rate ($u={10}^{-5}{s}^{-1}$), a high probability of proteasome cleavage (${p}_{i,j}=0.1$), and a fast rate of supply into the ER (${g}_{i}=0.08$ peptides s^−1^). In doing so, we could test how fast peptides can arrive at the surface of infected cells in a near-optimal scenario, establishing an approximate upper bound. The kinetics of the proteins contained in one virion over 9 hours post infection is shown in Fig. 9A. Each protein declines in abundance as it is degraded and converted into peptides. The resulting peptide presentation from a single infecting virion produced very low numbers for all peptides, with only the Gag peptide exceeding 1 copy on average (based on 300 independent simulations) within 9 hours. Therefore, the probability that a non-Gag peptide is presented from a single virion is small. However, if multiple virions enter a cell during infection, then this probability could increase and become immunologically relevant.

To investigate the impact of multiple virion infection and produce a more realistic quantification of the probability of infection, we used the well-known multiplicity of infection principle^44^, which quantifies the number of infecting virions using a Poisson distribution with mean 1, i.e. $N\sim Poisson(1)$. For this calculation, we used conditional probability, which allows the total probability to be calculated in terms of the probability of presentation for a given number of virions (see Methods). Accordingly, we calculated the total probability of presentation of a peptide from each HIV protein, over time, assuming Poisson-distributed virion numbers up to 5 (Fig. 9C,D). As *N* increases, the concentration of the proteins entering the cytoplasm increases, which means a larger number of peptides will be produced, increasing the probability that a given peptide would be presented. For all values of *N*, the Gag peptide is the most abundant on the cell surface. The total probability of Gag peptide presentation converges towards 0.63, equal to the probability that at least one virion infects the cell, i.e. P(N > 0). Crucially, we predict a greater than 50% chance that a Gag peptide will be presented by 3 hours post-infection, reconciling the observations in ref.^43^. Our model also predicted that an efficient Vpr peptide has a higher probability of presentation than an equivalently efficient Pol peptide. This may be because the Pol degradation rate we are using in this simulation is the same as the Gag-Pol polyprotein degradation rate (see Methods for more description). However, the Gag-Pol polyprotein is cleaved in to the enzymes integrase, reverse transcriptase and protease. The degradation of these smaller constituent proteins could be faster than that of the polyprotein, which will affect the timing and probability of presentation of the peptides cleaved from the Pol enzymes.

## Discussion

The aim of this study was to predict the kinetics of the cell surface abundance of viral peptides throughout a viral replication cycle within a single infected cell. In doing so, we have made use of several bioinformatics tools that provide a static snapshot of the sequence-dependent processes that determine whether a peptide becomes a T-cell epitope. By embedding these tools in a dynamic model, we have been able to analyse how time-dependent factors, such as protein abundance, influence whether a peptide will be presented on the surface of virus-infected cells. The abundance of a peptide in the ER is dependent upon its cytoplasmic proteasomal cleavage probability and rate of transport in to the ER, here described via the peptide affinity with TAP. A higher ER abundance, along with a high affinity to the MHC allele in question will result in a high cell surface abundance, and thus a higher probability of T-cell response. To highlight the impact of time-dependent protein abundance and to provide a better conceptual understanding of the antigen presentation pathway and the important rate processes involved, we specifically applied these concepts to construct a mechanistic model of HIV-1 peptide presentation. However, the approach adopted here could in principle be applied to any virus, providing time-course measurements, or better still, a dynamic model, of intracellular protein abundance are available.

We modelled both the presentation of peptides derived from the degradation of HIV-1 virion proteins, and those derived from the degradation of *de novo* synthesised HIV proteins during viral replication. Simulations of an *efficient* epitope for each HIV-1 protein predicted that Gag peptides would dominate at the cell surface. We used longer timescale (up to 72 hours post-infection) simulations of the model to compare peptide presentation by HLA alleles associated with control of HIV against HLA alleles associated with fast progression to AIDS. The machine learning algorithms used in the IEDB MHC-I prediction tools provide accurate predictions of epitopes when comparing peptide sequences from within the same protein. Therefore, in order to simulate the presentation of possible HIV peptides by different HLA alleles, we used the values of the peptide-MHCI affinity and proteasomal cleavage score, predicted by the IEDB MHC-I processing tool to infer relative parameter values for each peptide. A sensitivity analysis revealed that protein synthesis is more influential to cell surface peptide abundance than proteasomal degradation and the proteasomal cleavage probability *pc* is initially the most important peptide-specific parameter to cell surface abundance, with the sensitivity constant with time. However, the importance of the peptide-MHCI unbinding rate *u*, increases with time, becoming the most important parameter by 36 hours post infection, suggesting sustained peptide-MHCI presentation is dependent upon the complex stability.

For peptides produced during *de novo* synthesis the model predicts that all alleles analysed herein will present a combination of Gag and Nef peptides at early times following infection, but eventually, Gag peptides can be displaced by highly stable peptides. For instance, alleles that are not associated with long-term control of HIV present Vpr peptides at high average abundance (B*07:02, B*55:01 and B*35:03) in combination with high average presentation of Gag, whereas alleles that are associated with long-term control present Vpr in very low average abundance, preferring to present a combination of Gag and Nef, Pol or Env in high abundance. As previously mentioned, the Pol protein is the most highly conserved sequence in HIV-1, and so stable binding to Pol-derived peptides would naturally confer protection. The time-dependent nature of cell surface abundance naturally raises the question of whether peptides presented earlier or later are better vaccine targets.

In a previous study that used peptide binding predictors to compare HIV epitopes across HLA alleles^28^, it was shown that the controlling alleles HLA-B*58:01,-B*57:01 and -B*27:05 have an intrinsic preference for p24 Gag peptides, whilst non-controlling alleles HLA-B*35:03 and -B*53:01 have a preference for Nef peptides. When considering the top three best-binding epitopes from each HIV-1 protein, this distinction was offered as an explanation of why some HLA alleles are protective against HIV progression. In this study, where immunogenicity is explored using sequence binding preferences of HLA alleles in combination with protein kinetics and peptide processing steps, we predict that the controlling alleles considered by Borghans *et al*.^28^ present almost exclusively Gag peptides in high abundance at 24 hours post-infection, whereas B*35:03 has a more varied repertoire, including peptides from Pol, Nef and Env (Fig. 6). Furthermore, we predict that the non-controller B*18:01 does not sustain high cell surface abundance of Gag-derived peptides, with Pol-derived peptides displacing them at later times, providing further support for the need for sustained and varied presentation of Gag peptides for control. Our simulations, however, do not provide any further support to the suggestion that the presentation of Nef peptides is linked to fast HIV progression, but rather suggest that non-controlling alleles tend to present Vpr peptides in high abundance, and that this is less conducive to control.

Our analysis of the shorter timescale (up to 9 hours post infection) presentation of viral peptides originating from the infecting virions offers insights that are specifically not possible from peptide binding prediction alone. However, the importance of these earlier times should not be overlooked. We calculated the probability that at least one peptide would be presented, and found that there was a greater than 50% chance of an efficient Gag peptide being presented within 3 hours, explaining the observations of the Gag epitopes KF11 and KK10 within 3 hours post infection^43^. The T-cell responses to early peptides could strongly shape the eventual dominating TCR clonotypes. In this study, we did not attempt to simulate specific peptide sequences arising from the infecting virion, as the required stochastic treatment is computationally cumbersome, instead leaving a more detailed analysis to future work.

Whilst we used HIV-1 clade C in this study, our modelling methodology could also be applied to any HIV clade for which the full amino acid sequence is available. Naturally, changes in sequence would lead to changes in derived peptide sequences, which could both add and remove immunologically relevant peptides. Furthermore, changes in viral sequence could have an impact on the rates of transcription and translation of viral proteins, however these effects would be difficult to predict. As such, the rates used in our model are not clade-specific. Furthermore, as noted above, there is no reason that other viruses could not also be analysed using our approach, though the timing of protein accumulation and a quantification of virion composition would be required.

Unfortunately, very little experimental data exists that can quantitatively test the predictions made in this model. Quantitative data is difficult to gather experimentally due to the expense involved in these procedures, resulting from the large number of peptides which would need to be scanned, and the multiple MHC alleles the experiment would have to be repeated on^21^. Therefore, all of our efforts to compare with experimental observations are qualitative, but have established that known epitopes are indeed predicted to be presented in high abundance. Advances in mass spectrometry have enabled more comprehensive datasets to emerge for other viruses, such as LCMV^45^, which might be used to test the general applicability of this dynamic modelling strategy to predict immune recognition. More high throughput technologies, such as DNA barcoded peptide-MHC complexes^46^, could also help to identify the peptide specificities of T-cell receptors that are relevant for specific infections. Knowledge of the hierarchy and timing of the presentation of T-cell epitopes could be helpful in developing successful T-cell vaccines. Predictive models such as the one presented here are useful for helping to design experiments that provide a mechanistic understanding of immune recognition.

## Methods

### Prediction of HIV epitopes using bioinformatics tools

We used the MHC class I epitope prediction tools available from the IEDB (http://tools.iedb.org/processing/; version 2013-02-22). The tool combines the output of three separate predictions algorithms for proteasomal cleavage, TAP transport and MHC-I-peptide binding into a ‘Total Score’^24,47^. The Total Score is design to be proportional to the cell surface abundance of the peptide bound to an MHC I molecule. A Stabilised Matrix Method (SMM)^24,48,49^ is used in both the proteasomal cleavage predictions and TAP-peptide affinity predictions, where the assumption that each amino acid in a sequence contributes independently to MHC-I binding, is modified by including pair-wise interactions between the amino acids within the peptide. The peptide-MHC binding affinity predictions are provided by the NetMHCpan tool, which uses Artificial Neural Networks (ANN)^50^. The quality of these combined predictions is better than or equal to several methods that instead focus on MHC-I peptide binding in isolation^24,51^, such as BIMAS^22^. We applied the prediction tools to the consensus HIV-1 clade C sequence available at http://www.hiv.lanl.gov/content/index for each of the nine HIV-1 proteins. The sequence is used as an input to the tool, and the user can then select which HLA allele they want to predict the peptide binding affinities for.

### Deterministic simulation of the HIV-1 intracellular kinetics model

We built a dynamical systems model of HIV-1 intracellular kinetics by combining three existing models of HIV viral dynamics made up of systems of ODEs, Kim & Yin^29,52^, Reddy & Yin^18^, and Wang & LuHua^30^ (Table 1). The model by Kim & Yin^29^ describes the translation of full-length (*F*_*N*_) and successive splicing to produced singly-spliced (*S*_*N*_), and multiply-spliced (*M*_*N*_) HIV mRNA in the nucleus, the export of these transcripts, the translation of the HIV proteins Rev and Tat and their influence upon mRNA export and transcription rate respectively. Initially, *F*_*N*_ is transcribed at the basal cellular transcription rate, ${T}_{{C}_{b}}$; however, once copies of the Tat protein start appearing in the nucleus they can bind to the transactivation response element (TAR), a process which can be modelled according to Michaelis-Menten kinetics, using the equilibrium constant of Tat binding with TAR ${K}_{Tat}$. Full-length mRNA can be spliced to produce singly-spliced mRNA, which can then be spliced to produce multiply-spliced mRNA, with rate coefficients ${k}_{sp}^{F}={k}_{sp}^{S}$. Only *M*_*N*_ can be independently exported to the cytoplasm, where it can be translated to produce Rev and Tat, which can then be imported back in to the nucleus. The larger transcripts require Rev to bind to the Rev response element (RRE), and once a threshold number of Rev proteins have been bound ($i\ge Th$), the transcripts can then be exported. The number of Rev proteins bound to a single transcript can range from between $1\le i\le 12$. The binding of Rev to a transcript causes a delay in splicing of factor $d$. If a full-length transcript with *i* bound Rev proteins, $F{R}_{N}^{(i)}$, is spliced with rate coefficient $\mathrm{(1}-d){k}_{sp}$ this produces singly-spliced mRNA with *i* bound Rev proteins, $S{R}_{N}^{(i)}$. If $S{R}_{N}^{(i)}$ is spliced it produces multiply-spliced mRNA ${M}_{N}$ and *i* free Rev proteins. Once the threshold number $Th$ of bound Rev proteins have been reached, the transcripts can be exported to the cytoplasm with rate coefficient ${k}_{exp}$, where the Rev proteins instantaneously unbind, and are free to shuttle back in to the nucleus, with rate coefficient ${k}_{imp}$.

**Table 1:** Equations describing intracellular kinetics of HIV transcripts and the Rev and Tat proteins. All equations and parameters are taken from Kim & Yin^29^.

**Description**	**Equation**
Full-length mRNA (*F*_*N*_) in the nucleus	$\frac{d[{F}_{N}]}{dt}={T}_{{C}_{b}}+{T}_{{C}_{add}}\frac{{K}_{Tat}[{T}_{N}]}{1+{K}_{Tat}[{T}_{N}]}pv+{k}_{d}^{\mathrm{(1)}}[F{R}_{N}^{\mathrm{(1)}}]-({k}_{sp}^{F}+{k}_{deg,N}^{RNA}+{k}_{a}^{\mathrm{(1)}}S[{R}_{N}])[{F}_{N}]$
*F*_*N*_ with *i* bound Rev proteins $F{R}_{N}^{(i)}$	$\frac{d[F{R}_{N}^{(i)}]}{dt}={k}_{a}^{(i)}[{R}_{N}][F{R}_{N}^{(i-\mathrm{1)}}]+{k}_{d}^{(i+\mathrm{1)}}[F{R}_{N}^{(i+\mathrm{1)}}]$
	$$-({k}_{d}^{(i)}+{k}_{a}^{(i+\mathrm{1)}}[{R}_{N}]+{k}_{exp}^{(F,i)}+\mathrm{(1}-{d}^{F,(i)}){k}_{sp}^{F}+{k}_{deg,N}^{RNA})[F{R}_{N}^{(i)}]$$
Singly-spliced nuclear mRNA, *S*_*N*_	$\frac{d[{S}_{N}]}{dt}={k}_{sp}^{F}[{F}_{N}]+{k}_{d}^{\mathrm{(1)}}[S{R}_{N}^{\mathrm{(1)}}]-({k}_{sp}^{S}+{k}_{deg,N}^{RNA}+{k}_{a}^{\mathrm{(1)}}[{R}_{N}])[{S}_{N}]$
*S*_*N*_, with *i* bound Rev proteins, $S{R}_{N}^{(i)}$	$\frac{d[S{R}_{N}^{(i)}]}{dt}={k}_{a}^{(i)}[{R}_{N}][S{R}_{N}^{(i-\mathrm{1)}}]+{k}_{d}^{(i+\mathrm{1)}}[S{R}_{N}^{(i+\mathrm{1)}}]+\mathrm{(1}-{d}^{F,(i)}){k}_{sp}^{F}[F{R}_{N}^{(i)}]$
	$$-({k}_{d}^{(i)}+{k}_{a}^{(i+\mathrm{1)}}[RN]+{k}_{exp}^{S,(i)}+\mathrm{(1}-{d}^{S,(i)}){k}_{sp}^{S}+{k}_{deg,N}^{RNA})[S{R}_{n}^{(i)}]$$
Multiply-spliced nuclear mRNA, *M*_*N*_	$\frac{d[{M}_{N}]}{dt}={k}_{sp}^{S}[{S}_{N}]+{\sum }_{i\mathrm{=1}}^{sn}\mathrm{((1}-{d}^{S,(i)}){k}_{sp}^{S}[S{R}_{N}^{(i)}])-({k}_{exp}^{M}+{k}_{deg,N}^{RNA})[{M}_{N}]$
Cytoplasmic multiply-spliced mRNA, *M*_*C*_	$\frac{d[{M}_{C}]}{dt}={k}_{exp}^{M}[{M}_{N}]-{k}_{deg,C}^{RNA}[{M}_{C}]$
Cytoplasmic full-length mRNA, *F*_*C*_	$\frac{d[{F}_{C}]}{dt}={\sum }_{i\mathrm{=1}}^{sn}({k}_{exp}^{F,(i)}[F{R}_{N}^{(i)}])-{k}_{deg,C}^{RNA}[{F}_{C}]-\mathrm{(2}\times \frac{dVirion}{dt})$
Cytoplasmic singly-spliced mRNA, *S*_*C*_	$\frac{d[{S}_{C}]}{dt}={\sum }_{i\mathrm{=1}}^{sn}({k}_{exp}^{S,(i)}[S{R}_{N}^{(i)}])-{k}_{deg,C}^{RNA}[{S}_{C}]$
Cytoplasmic Rev protein, *R*_*C*_	$\frac{d[{R}_{C}]}{dt}={f}_{Rev}\cdot Tr\cdot {f}_{rev}^{M}[{M}_{C}]+{k}_{exp}^{R}[{R}_{N}]+{\sum }_{i\mathrm{=1}}^{sn}(i\cdot ({k}_{exp}^{F,(i)}[F{R}_{N}^{(i)}]+{k}_{exp}^{S,(i)}[S{R}_{N}^{(i)}])-({k}_{imp}^{R}+{k}_{deg,C}^{R})[{R}_{C}]$
Nuclear Rev protein, *R*_*N*_	$\frac{d[{R}_{N}]}{dt}={k}_{imp}^{R}[{R}_{C}]+{\sum }_{i\mathrm{=1}}^{sn}({k}_{d}^{(i)}\cdot ([F{R}_{N}^{(i)}]+[S{R}_{N}^{(i)}]))+{\sum }_{i\mathrm{=1}}^{sn}(i\cdot {k}_{deg,N}^{RNA}([F{R}_{N}^{(i)}]+[S{R}_{N}^{(i)}]))$
	$$+{\sum }_{i\mathrm{=1}}^{sn}(\mathrm{(1}-{d}^{S,(i)}){k}_{sp}^{S,(i)}[S{R}_{N}^{(i)}])-({\sum }_{i\mathrm{=1}}^{sn}({k}_{a}^{(i)}([F{R}_{N}^{(i)}]+[S{R}_{N}^{(i)}])+{k}_{exp}^{R}+{k}_{deg,N}^{R})[{R}_{N}]$$
Cytoplasmic Tat protein, ${T}_{C}$	$\frac{d[{T}_{C}]}{dt}={f}_{Tat}\cdot Tr\dot{(}{f}_{tat}^{S}[{S}_{C}]+{f}_{tat}^{M}[{M}_{C}])+{k}_{exp}^{T}[{T}_{N}]-({k}_{imp}^{T}+{k}_{deg,C}^{T})[{T}_{C}]$
Nuclear Tat protein, ${T}_{N}$	$\frac{d[{T}_{N}]}{dt}={k}_{imp}^{T}[{T}_{C}]-({k}_{exp}^{T}+{k}_{deg,N}^{T})[{T}_{N}]$

**Figure 10:**
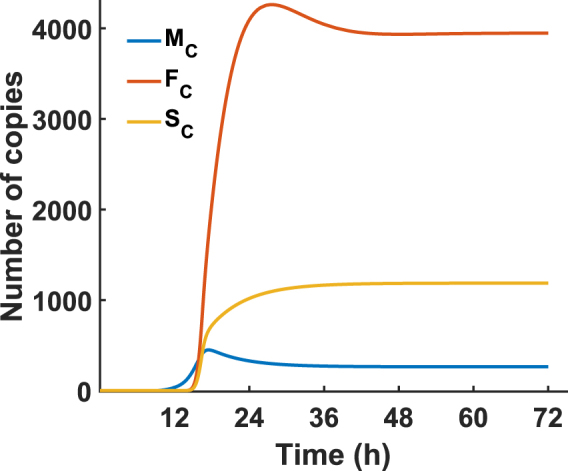
Simulated HIV mRNA. The model in Table 0 was used to simulate full-length (*F*_*C*_), singly-spliced (*S*_*C*_) and multiply-spliced (*M*_*C*_) mRNA copies in the cytoplasm. The full-length cytoplasmic mRNA (*F*_*C*_) reaches a steady state level of 3,900 copies, which agrees with the experimentally measured average^53^.

The final term on the right-hand side of the equation for *F*_*C*_ does not originate from the Kim & Yin model^29^, but instead comes from the Wang & LuHua^30^ model. This term, $-2\times \frac{dVirion}{dt}$ accounts for the export of full-length mRNA to the cell membrane for incorporation in to the budding virion, and ensures the steady-state level of full-length mRNA in the cytoplasm remains at the experimental average of 3,900^53^. The kinetics of *Virion* are described in Equation Set 2 and all parameter values are given in Supplementary Table S1. The resulting cytoplasmic mRNA kinetics are shown in Fig. 10.

To model the dynamics of the important structural proteins, Gag, GagPol and Env, we use the model presented by Reddy and Yin^18^ for the synthesis and degradation steps (Table 2), although we use the mRNA levels produced in the Kim & Yin^29^ model and the budding rate from Wang & LuHua^30^. The kinetics of the Vif protein are also provided by Wang & LuHua^30^. All parameters used here are taken from Wang & LuHua^30^ or Reddy and Yin^18^. Full-length mRNA encodes for the important structural proteins Gag and GagPol, with are translated with rate coefficients ${f}_{Gag}\cdot Tr$ and ${f}_{GagPol}\cdot Tr$ respectively, where ${f}_{Prot}$ are the translation fractions and *Tr* is the translation rate. Each protein degrades with rate coefficient ${k}_{Prot}$ and is exported to the cell membrane for budding with rate constant ${k}_{bud}$.

**Table 2:** Equations describing intracellular kinetics of HIV proteins Gag, GagPol, Env and Vif. All equations and parameters are taken from Wang & LuHua^30^ or Reddy & Yin^18^.

Description	Equation
Gag kinetics	$\frac{d[Gag]}{dt}={f}_{Gag}\cdot Tr[{F}_{C}]-{k}_{Gag}[Gag]-{k}_{bud}[Gag]$
GagPol kinetics	$\frac{d[GagPol]}{dt}={f}_{GagPol}\cdot Tr[{F}_{C}]-{k}_{GagPol}[GagPol]-{k}_{bud}[GagPol]$
Env kinetics	$\frac{d[Env]}{dt}={f}_{Env}\cdot Tr[{S}_{C}]-{k}_{Env}[Env]-{k}_{bud}[Env]$
Vif kinetics	$\frac{d[Vif]}{dt}={f}_{Vif}\cdot Tr[{S}_{C}]-{k}_{Vif}[Vif]-{k}_{bud}[Vif]$
Virion kinetics	$\frac{dVirion}{dt}=\frac{{k}_{bud}[Gag]}{{n}_{Gag,virion}}$

For the remaining HIV-1 proteins, Vpr, Vpu and Nef, not included in any of the existing three models, we used the general equation for the protein kinetics in the cytoplasm, i.e. $d[{{\rm{Prot}}}_{j}]/dt={f}_{j}\cdot Tr[mRNA]-{k}_{j}[{{\rm{Prot}}}_{j}]-{k}_{bud}[{{\rm{Prot}}}_{j}]$, using experimentally measured values for ${k}_{j}$ when available, and using the same value of ${k}_{{\rm{bud}}}$ as given in Wang & LuHua^30^. The synthesis fractions ${f}_{j}$ were set so that the amount of protein being incorporated in to one virion via the budding term was equal to the experimentally determined concentration of the protein found in a HIV virion. All three models use parameters taken from the literature and compare their results with experimental data where available. Combining these three models, along with experimental measurements of missing rates such as protein half-lives, produces an almost complete model of all HIV intracellular kinetics, in terms of viral protein dynamics. This model can be updated as more experimental data becomes available and the parameters values can be refined.

### Quantifying peptide-dependent rates

To predict the differences in the cell surface presentation of HIV peptides by controlling and non-controlling alleles using this model, we required methods for quantifying the rates of peptide unbinding of MHC-I, proteasomal cleavage and peptide degradation in the cytoplasm. We applied the following methods to the consensus sequences of HIV clade C from the Los Alamos National Laboratory (LANL) HIV database to obtain relative values for peptide unbinding, proteasomal cleavage and peptide degradation. We decided to ignore the impact of peptide-dependent TAP binding in this study, as the sensitivity analysis suggested it to have only a minor role in shaping cell surface presentation on MHC-I molecules (Fig. 5). We required that the IEDB predicted peptide-MHC affinity values be comparable between alleles. Therefore, we rescaled the IC50 affinity values according to the method in ref.^54^ and used in similar studies to this^28,55^. We acquired the predicted IC50 values for the peptides from the *Mycobacterium Tuberculosis* proteome to obtain a dataset of over 500,000 partially overlapping natural peptides. For each allele studied here we obtained three separate datasets for the 9mers, 10mers and 11mers. We then combined the three datasets and took the top 1% of binders as the IC50 threshold for binding peptides for each allele. The rescaling method described in ref.^54^ normalises each IC50 by dividing by the threshold IC50 value. However, for our purposes we require a rescaled IC50 value that is still in units of $nM$. Therefore, we arbitrarily chose one allele as the reference allele and then rescaled the predicted IC50 values relative to that allele. The reference allele was chosen to be HLA-B*58:01, and its threshold affinity as determined using the method described above is denoted ${I}_{B58}$. When rescaling the predicted IC50 values for say HLA-B*57:01, we would multiply the IC50 value by the ratio of the threshold of B*58:01 to the threshold of B*57:01. Therefore, for allele *a*, the rescaled IC50 values are calculated as follows: $IC{50}_{a}^{R}=IC{50}_{a}{I}_{B58}{I}_{a}$, where $IC{50}_{a}^{R}$ is the rescaled IC50 of allele *a*, $IC{50}_{a}$ is the original IC50 and ${I}_{a}$ is the rescale threshold of allele *a*.

#### Peptide unbinding from MHC-I

While there exist prediction methods for peptide-MHC stability directly, such as NetMHCstab and NetMHCstabpan, none have been calibrated to cover the HLA alleles that are of interest in this study. Therefore, we based our quantification of peptide off-rate on the more common affinity-based methods that are suggested in the IEDB MHC-I processing tool, but note that this will likely induce some degree of inaccuracy^6^. We first assumed that the predicted IC_50_ value for each peptide binding to an MHC allele is approximately equal to the dissociation constant^56–59^, ${K}_{d}$, where ${K}_{d}=u/{b}_{P}$ where *u* and *b*_*P*_ are the peptide MHC unbinding and binding rates respectively. We then assumed that the binding rate is constant for each peptide, as experimental evidence shows that there is much less variation in HIV peptide binding rates than in the peptide off-rates^60^, and used the value 219 M^−1^ s^−1^ measured in ref.^61^. This enabled us to estimate the unbinding rate of each peptide as $u={K}_{d}{b}_{P}$.

#### Proteasomal cleavage

The IEDB tool outputs a cleavage score for each peptide that is proportional to the logarithm of the amount of peptide generated from the cleavage of the peptides C-terminal. We converted this to a relative cleavage probability for each peptide sequence. First, we established a range in which these relative probabilities should lie. The well known peptide SIINFEKL, or an N-terminally extended version of it, was measured to be produced via degradation of the OVA protein 6–8% of the time it degrades^62^. The range of the IEDB predicted relative abundances were in the range [1, 80]. Therefore, we scaled these values down by a factor 1000, producing cleavage scores in the range [0, 0.08], consistent with SIINFEKL being produced from OVA degradation with high probability.

### The peptide filtering model for MHC-I antigen presentation

The peptide filtering model is a dynamical systems description of peptide-MHC binding and presentation at the cell surface^33^. The equations are reproduced in Supplementary Equation Set S3. Here, we provide a short description of how the underlying biochemistry is modelled. A peptide *P*_*i*_ is supplied to the ER with supply rate coefficient *g*_*i*_, where it can either be degraded with rate constant ${d}_{P}$ or bind to an MHC molecule $M$ with rate ${b}_{P}$, or an MHC-tapasin complex with a higher rate ${c}_{P}$. Tapasin is a chaperone molecule that binds to the peptide loading complex and ensures that only high affinity peptides are presented on the cell surface. Tapasin and MHC are supplied to the ER with rate coefficients ${g}_{T}$ and ${g}_{M}$ and degrade with rate coefficients ${d}_{T}$ and ${d}_{M}$ respectively. The peptide-MHC complex $M{P}_{i}$ unbinds with the peptide sequence-dependent rate constant *u*_*i*_, whilst the peptide unbinds the peptide-MHC-tapasin complex with a faster rate coefficient $q\cdot {u}_{i}$ (*q* > 1). MHC-tapasin complexes dissociate with rate *u*_*T*_, whereas in the presence of the peptide, tapasin unbinds the peptide-MHC-tapasin complex with an increased unbinding rate coefficient ${u}_{T}\cdot v$. Finally, a peptide-MHC complex can egress to the cell surface with rate coefficient *e*, where the peptide again unbinds from MHC-I with rate *u*_*i*_.

In all, there are five peptide-dependent equations. Therefore, if the IEDB prediction tool predicts $n$ peptides with an IC_50_ less than 500 nM, then there will be approximately $5n$ equations in the system. In some cases the IEDB tool predicts that hundreds of peptides from the entire HIV genome will bind to the HLA allele in question, resulting in a very large system which must be solved numerically, especially in the case of the deterministic intracellular kinetics model. As the resulting model has stiff kinetics, we used Matlab’s ode15s integrator. We provide the Jacobian matrix for the system as a sparse matrix to prevent the need for the solver to approximate the Jacobian numerically, which in this case would create an unnecessary memory burden.

#### Self-peptides

Viral peptides compete not only with each other but also with self-peptides for MHCI binding and presentation. Self-peptides originate from native host proteins and can also be presented on the cell surface, however they should not initiate a T-cell response due to negative selection of self-reactive T-cells. Just like the viral peptides, these self-peptides will have a range of proteasomal cleavage probabilities, ER supply rates and MHCI binding rates. However, there are too many self-peptides to represent explicitly in the model. Therefore, to model the impact of the competition of these self-peptides we represented the self-peptides by four additional peptides in the simulation. These four peptides were given a range of unbinding and supply rates (see Table S5 for parameters). Each different MHC allele will only bind a small subsection of the ER peptidome with high affinity, and so the self-peptides with a medium unbinding rate (1 × 10^−3^ *s* ^−1^) were assumed to make up the majority of the peptides being transported in to the ER and were allocated a large fraction of the total supply rate. TAP binds between 2–5 peptides per second, and there are approximately 10,000 copies of TAP per cell^20^. We assigned the total rate of TAP transport of self-peptides to be the lower end of this range (20,000 peptides per second) to maximise viral peptide presentation. The kinetics of the self-peptides are given by are identical to those described for peptides in Equation S3.

### Sensitivity analysis

We performed a sensitivity analysis on a small subset of the model parameters for the deterministic system of HIV intracellular kinetics, using the SUNDIALS CVODES^63^ forward sensitivity analysis (FSA) in MATLAB, with the aim of comparing the importance of different parameters which are known to influence cell surface abundance: peptide-MHC binding rate *b*_*P*_, peptide-MHCI-tapasin binding rate *c*_*P*_, peptide-MHCI unbinding rate *u*_*i*_, protein translation (where here we used the translation fractions of each protein *f*_*j*_), protein degradation rate *k*_*j*_, proteasomal cleavage probability $p{c}_{i}$ and peptide ER supply rate *g*_*i*_.

The sensitivity of a system of nonlinear first order ODEs $\dot{x}=f(t,x,\theta )$ with respect to the k^th^ parameter ${\theta }_{k}$ is computed as the partial derivative of that function with respect to the parameter:

2 $${\dot{s}}_{i}=\frac{d}{dt}(\frac{{\rm{\partial }}{x}_{i}}{{\rm{\partial }}{\theta }_{k}})=\sum _{m=1}^{{x}_{dim}}(\frac{{\rm{\partial }}{\dot{x}}_{i}}{{\rm{\partial }}{x}_{m}}\frac{{\rm{\partial }}{x}_{m}}{{\rm{\partial }}{\theta }_{k}})+\frac{{\rm{\partial }}{\dot{x}}_{i}}{{\rm{\partial }}{\theta }_{k}}$$

The CVODES FSA approximates ${\dot{s}}_{i}$ by a centred difference quotient. Both the sensitivities and ODE systems are solved simultaneously, to provide the time dependent parameter sensitivity. We applied CVODES to the output of our combined model by considering ${x}_{i}={[M{P}_{i}]}_{{\rm{cs}}}$. To normalize each time-dependent sensitivity coefficient, we multiplied $\frac{\partial {[M{P}_{i}]}_{{\rm{cs}}}}{\partial {\theta }_{k}}$ by ${\theta }_{{k}_{0}}/[M{P}_{i}{]}_{{\rm{cs}}}(t)$, as described in ref.^37^.

### Virion model

The deterministic model of HIV intracellular replication describes the concentration of each species as a continuous variable, and so in fact describes the average of the population. The assumption that reactions occur at a constant rate is value when considering large numbers of molecules of each interacting species, as is being simulated in the large combined model described above. However, when simulating very small populations of molecules however, the reacting molecules will not come in to contact at a constant rate and a deterministic model that uses constant reaction rates would not describe this system very well as one would expect to see a large variation from the average behaviour of a larger system. The discrete stochastic formulation considers the exact number of molecules present in the system at that time and the probability of each possible reaction occurring within a certain time interval. This assumes that the probability of the reaction $$A+B\mathop{\rightharpoonup }\limits^{k}C$$ firing in time interval $$[t,t+dt)$$ is exponentially distributed with mean $$A(t)B(t)dt$$.

For simulating cell surface presentation of virion-derived peptides, we used a stochastic model equivalent to the equations of Table S3, which uses elementary chemical reactions to describes the copy numbers of each molecular species as discrete variables. The complete set of reactions is given in Table 3. The peptides are produced as described in equation 1, and connected up to the MHC peptide filtering model using the supply term ${g}_{i}{[{P}_{i}]}_{{\rm{cyt}}}$ as before.

**Table 3:** Chemical reaction network model of peptide filtering. The reactions are extended from^33^ to include the degradation of protein *j*, and proteasomal cleavage and ER translocation from the cytosol of peptide *i*. The superscripts denote the compartment containing the molecules (cyt - cytoplasm; cs - cell surface), with no superscript denoting ER. The superscript is omitted from Prot_*j*_, which is always in the cytoplasm.

${{\rm{Prot}}}_{j}\,\mathop{\longrightarrow }\limits^{{k}_{j}}\,0/$	$0/\,\underset{{d}_{{P}_{C}}}{\overset{p{c}_{i}\mathrm{.}{k}_{j}}{{\rightleftharpoons}}}\,{P}_{i}^{{\rm{cyt}}}$
${P}_{i}^{{\rm{cyt}}}\,\mathop{\longrightarrow }\limits^{{g}_{i}}\,P$	${P}_{i}\,\mathop{\longrightarrow }\limits^{{d}_{{P}_{ER}}}\,0/$
$0/\,\underset{{d}_{M}}{\overset{{g}_{M}}{{\rightleftharpoons}}}\,M$	$0/\,\underset{{d}_{T}}{\overset{{g}_{T}}{{\rightleftharpoons}}}\,T$
${P}_{i}+M\,\underset{{u}_{i}}{\overset{{b}_{P}}{\rightleftharpoons }}\,M{P}_{i}$	${P}_{i}+MT\,\underset{q\mathrm{.}{u}_{i}}{\overset{{c}_{P}}{\rightleftharpoons }}\,TM{P}_{i}$
$M{P}_{i}\,\mathop{\longrightarrow }\limits^{e}\,M{P}_{i}^{{\rm{cs}}}$	$M{P}_{i}^{{\rm{cs}}}\,\mathop{\longrightarrow }\limits^{{u}_{i}}\,{M}^{{\rm{cs}}}+{P}_{i}^{{\rm{cs}}}$

To perform the stochastic simulations, we used the Visual GEC software http://research.microsoft.com/gec, which uses Gillespie’s stochastic simulation algorithm^64^ (SSA) to simulate chemical reaction networks. As the algorithm is stochastic, each trajectory produced can only be considered as one sample from a distribution of samples. Therefore, we always used 300 independent trajectories, and computed statistics based on these such as the mean copy number, or the probability of there being at least one copy.

### Calculating overall presentation using conditional probability

Suppose we denote by $S(t)$ the surface presentation at time *t*. Conditional probability enables us to compute the total probability of presentation by separating out the influence of the number of infecting virions *N*. Accordingly,

3 $$P(S(t)\ge \mathrm{1)}=\sum _{i\mathrm{=0}}^{\infty }P(S(t)\ge \mathrm{1|}N=i\mathrm{).}P(N=i)$$

By assuming that *N* is Poisson distributed with mean 1, we can easily combine simulations of the combined model for different numbers of virions. In the calculations of Fig. 9D, we included simulations up to 5 virions, which covers 99.9% of the probability mass for a Poisson with mean 1.

## Electronic supplementary material

Supplementary Information
