# Supplementary material for: Host genotype and time dependent antigen presentation of viral peptides: predictions from theory
Source: Sci Rep. 2017 Oct 30;7:14367. doi: 10.1038/s41598-017-14415-8 (PMC5662608; doi:10.1038/s41598-017-14415-8)
Supplement: Supplementary file 1 — Supplementary Information [file 41598_2017_14415_MOESM1_ESM.pdf]

Supplementary Information

Host genotype and time dependent antigen presentation of viral peptides: predictions from theory

R Charlotte Eccleston[1,2], Peter V Coveney[1,2], and Neil Dalchau[3,*]

[1]Centre for Computational Science, Department of Chemistry, University College London, London, WC1H 0AJ, UK
[2]CoMPLEX, University College London, London, WC1E 6BT, UK
[3]Microsoft Research, Cambridge, CB1 2FB, UK
[*]ndalchau@microsoft.com

Parameter	Value	References
Basal transcription rate, T_{C_b}	4.1667×10^{-3} transcripts s^{-1}	1, 2
Increase in Transcription by Tat Transactivation, $T_{C_{add}}$	0.4125 transcripts s^{-1}	3–5
Equilibrium dissociation constant of Tat with TAR, K_{Tat}	5.2453×10^{-5} molecule^{-1}	28.57 μM^{-1}6
Splicing rate constants, $k^F_{sp} = k^S_{sp}$	6.95×10^{-4} molecule^{-1} s^{-1}	1, 2, 7
Rate of nuclear export $k^{F,(i)}_{exp} = k^{S,(i)}_{exp} = k^M_{exp} = k^R_{exp}, k^T_{exp} = 0$	5.7833×10^{-4} s^{-1}	8
Rate of Translation k_{Trans}	0.075 s^{-1}	1, 2, 9
Fraction of Full-Length mRNA encoding Gag f_{Gag}	0.95	2
Fraction of Full-Length mRNA encoding Gag-Pol f_{GagPol}	0.05	2
Fraction of Singly-spliced mRNA encoding Env f_{Env}	0.15	2
Fraction of Multiply-spliced mRNA encoding Rev f^M_{Rev}	0.19	1
Probability that Rev mRNA will encoding Rev f_{Rev}	0.5	1
Fraction of Multiply-spliced mRNA encoding Tat mRNA f^M_{Tat}	0.05	1
Fraction of Singly-spliced mRNA encoding Tat mRNA f^S_{Tat}	0.05	1
Probability that Tat mRNA encodes for Tat f_{Tat}	1.0	1
Splicing Delay Factor Due to Rev $d^{F,(i)} = d^{S,(i)}$	0.8	1
Nuclear Import Rate Constant $k^T_{imp} = k^R_{imp}$	$5.7833 \times 10^{-3} s^{-1}$	1, 10
Cytoplasmic degradation rate for Rev, $k_{Rev,C}$	$4.833 \times 10^{-6} s^{-1}$	1
Nuclear degradation rate for Rev, $k_{Rev,N}$	$1.2 \times 10^{-5} s^{-1}$	1
Degradation rate for Tat, $k_{Tat,C} = k_{Tat,N}$	$4.278 \times 10^{-5} s^{-1}$	1
Cytoplasmic degradation rate for gp120 (Env Precursor) $k_{gp120,C}$	$5.55 \times 10^{-6} s^{-1}$	2
Association constant for Rev with RRE, $k^{(1)}_a$, $k^{(i)}_a$	0.0132 molecule^{-1} s^{-1}, 0.0233 molecule^{-1} s^{-1}	1
Dissociation constant for Rev with RRE, $k^{(1)}_d$, $k^{(i)}_d$	3×10^{-5} molecule^{-1} s^{-1}, 0.038 molecule^{-1} s^{-1}	1
Budding rate k_{bud}	0.08 h^{-1}	6
Protein degradation of eukaryotic proteins $k_{\deg}$	1.083×10^{-4} s^{-1}	2
Fraction of Singly-spliced mRNA encoding Vif f_{Vif}	0.1	N/A
Fraction of Singly-spliced mRNA encoding Vpr f_{Vpr}	0.27	N/A
Fraction of Singly-spliced mRNA encoding Vpu f_{Vpu}	0.1	N/A
Fraction of Multiply-spliced mRNA encoding Nef f_{Nef}	0.5	N/A
Degradation rate of Gag, k_{Gag}	0.1054 h^{-1}	2
Degradation rate of GagPol, k_{Pol}	0.1159 h^{-1}	N/A
Degradation rate of Vpr, k_{Vpr}	0.0346 h^{-1}	11
Degradation rate of Env, k_{Env}	0.02 h^{-1}	2
Degradation rate Vpu, k_{Vpu}	0.086 h^{-1}	N/A
Copies of Gag per virion, $n_{Gag,virion}$	4900	12

Supplementary Table S1. HIV-1 Intracellular Kinetics Model Parameters. These parameters are used in the equations described in Table 1 & 2.

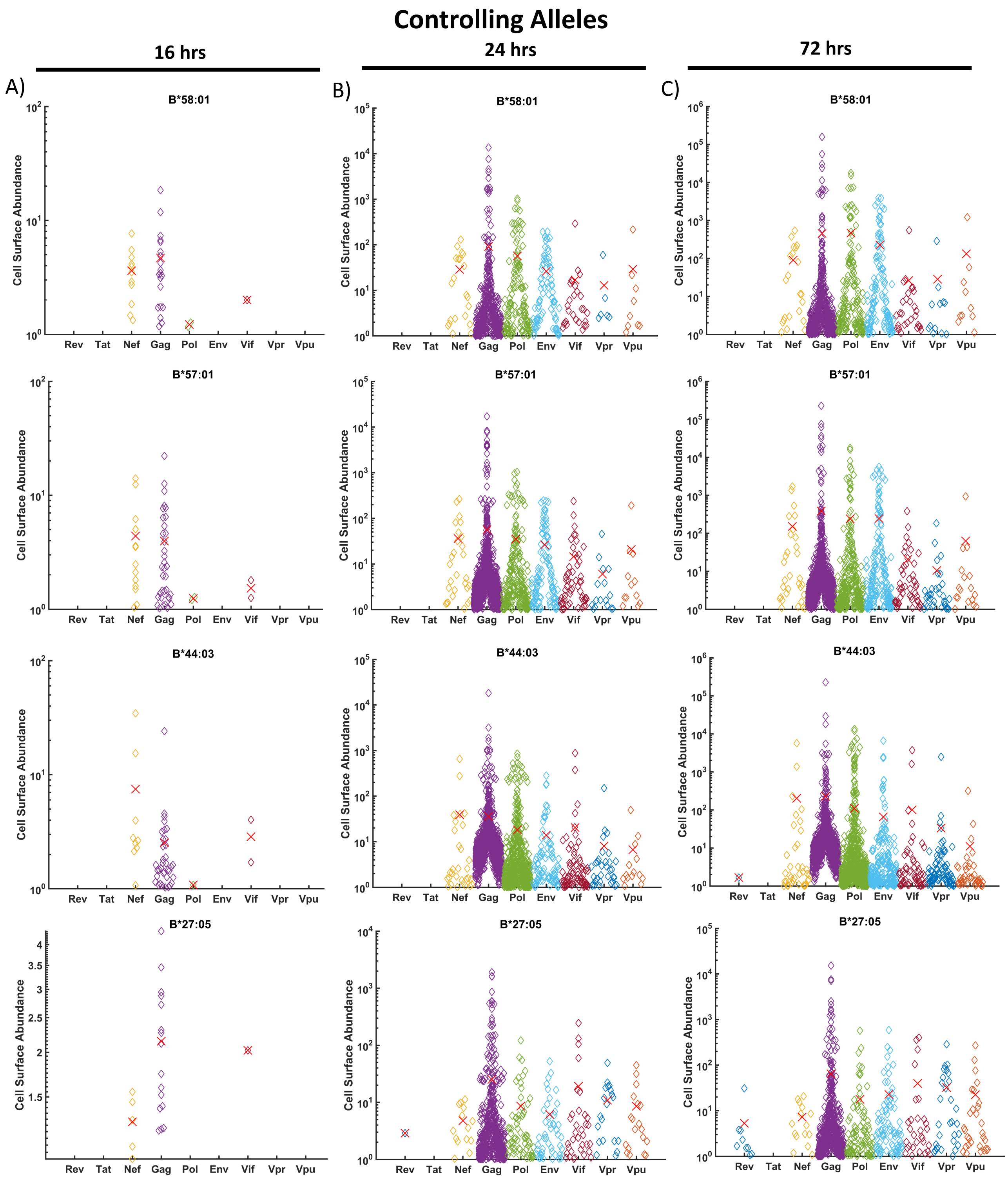

Supplementary Figure S1. Controlling alleles all demonstrate sustained Gag peptide presentation, and/or combined Gag and Pol peptide presentation at later times post infection We predicted the cell surface abundance of HIV-1 peptides by controlling alleles, and analysed the top 12 most abundant peptides at 16 h, 24 h and 72 h post-infection, where the bars are coloured by protein. All controlling alleles presented several Gag peptides by 16 h, with the number of Gag peptides increasing by 24 h post-infection. The presentation of these Gag peptides at high abundance is sustained up to 72 h post-infection.

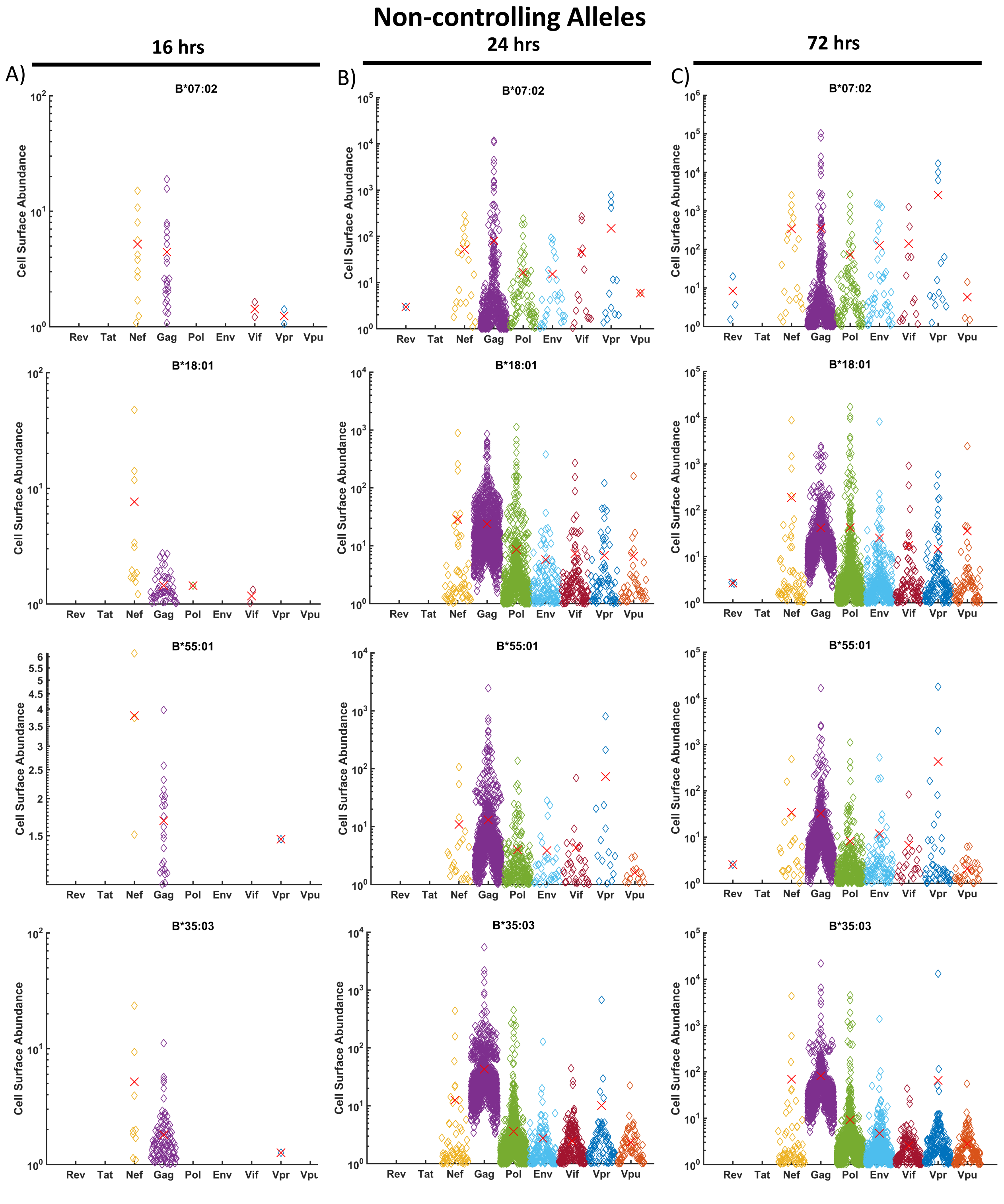

Supplementary Figure S2. Controlling alleles all demonstrate sustained Gag peptide presentation, and/or combined Gag and Pol peptide presentation at later times post infection We predicted the cell surface abundance of HIV-1 peptides by controlling alleles, and analysed the top 12 most abundant peptides at 16 h, 24 h and 72 h post-infection, where the bars are coloured by protein. All controlling alleles presented several Gag peptides by 16 h, with the number of Gag peptides increasing by 24 h post-infection. The presentation of these Gag peptides at high abundance is sustained up to 72 h post-infection.

Sequence	Protein	Position	Allele	Ref	Model (16 h)	Model (24 h)	Model (72 h)
KRWIILGLNK	p24	Gag (263-272)	HLA-B*27:05	13	2	1601	15027
TSTLQEQIAW	p24	Gag (240-249)	HLA-B*57:01/-B*58:01	14	11/12	7569/8262	55634/75657
KAFSPEVIPMF	p24	Gag (162-172)	HLA-B*57:01/-B*58:01	15	4/6	1791/3680	6629/19771
ISPRTLNAW	p24	Gag (147-155)	HLA-B*57:01/-B*58:01	16	0/6	1348/4179	5085/31567
IAMESIVIW	RT	Pol (530-538)	HLA-B*57:01/-B*58:01	17	0/0	1028/1035	17777/17719
AEQATQDVKNW	p24	Gag (306-316)	HLA-B*44:03	18	24	1819	22613

Supplementary Table S2. Known HIV-1 T cell epitopes and their predicted abundances at 16, 24 and 72 hours post-infection.

Description	Equation
MHC class I:	$\frac{d[M]}{dt} = g_M - d_M[M] - b_T[M][T] + u_T[MT] + u_i[MP_i] - b_P[M][P_i]$
Tapasin:	$\frac{d[T]}{dt} = g_T - d_T[T] - b_T[M][T] + u_T[MT] + u_T \cdot v[MTP_i]$
Peptide:	$\frac{d[P_i]}{dt} = g_i - d_P[P_i] - b_P[M][P_i] + u_i[MP_i] - c_P[MT][P_i] + q \cdot u_i[MTP_i]$
MHC-tapasin:	$\frac{d[MT]}{dt} = b_T[M][T] - u_T[MT] - c_P[MT][P_i] + q \cdot u_i[MTP_i]$
MHC-tapasin-peptide complexes:	$\frac{d[MTP_i]}{dt} = c_P[MT][P_i] - q \cdot u_i[MTP_i] - u_T \cdot v[MTP_i]$
MHC-peptide complexes:	$\frac{d[MP_i]}{dt} = b_P[M][P_i] - u_i[MP_i] + u_T \cdot v[MTP_i] - e[MP_i]$
Egressed MHC-peptide:	$\frac{d[MP_i]_{\text{cs}}}{dt} = e[MP_i] - u_i[MP_i]_{\text{cs}}$

Supplementary Table S3. Rate equations for the MHC class I pathway. The original peptide filtering model [19] is a two-compartment model describing the concentrations of peptide i (P_i) in complex with MHC-I (M) or tapasin-bound MHC-I (MT) in the ER and the cell surface. The concentrations at the cell surface are labelled with a subscript cs, while all ER concentrations are unlabelled. The model equations shown here were extended with an additional equation for cytoplasmic peptide, as described in the main text, in addition to the virion equations (Table 1 and 2). Accordingly, the parameter g_i listed here is replaced with a term $g_i[P_i]_{\text{cyt}}$ to complete the model of HIV infection.

Parameter	Description	Value
g_T	Tapasin supply	1505 molecules s^{-1}
d_T	Tapasin degradation	1.726×10^{-3} s^{-1}
g_M	MHC supply	150.5 molecules s^{-1}
d_M	MHC degradation	7.989×10^{-5} s^{-1}
d_{Me}	Cell surface MHC degradation	9.329×10^{-5} s^{-1}
d_P	Peptide degradation	0.13 s^{-1}
b_T	Tapasin-MHC binding	1.663×10^{-9} molecule^{-1} s^{-1}
u_T	Tapasin-MHC unbinding	1.185×10^{-6} s^{-1}
b_P	Binding of peptide to MHC	5×10^{-9} molecule^{-1} s^{-1}
c_P	Peptide binding to tapasin-MHC	$8.303 \times 10^{-8}\text{molecule}^{-1}$ s^{-1}
q	Effect of tapasin on peptide-MHC unbinding	2.104×10^{4}
v	Effect of peptide on tapasin-MHC unbinding	936.3
e	Egression of peptide-MHC	0.1142 s^{-1}

Supplementary Table S4. Peptide filtering model parameters. Values are as reported in Ref. 19.

Parameter	Unbinding rate (s^{-1})	Fraction of total supply
Self-peptide-MHC unbinding very high	1×10^{-2}	0.05
Self-peptide-MHC unbinding high	1×10^{-3}	98.5
Self-peptide-MHC unbinding medium	1×10^{-4}	0.05
Self-peptide-MHC unbinding low	1×10^{-5}	0.05

Supplementary Table S5. Self-peptide parameters.

Supplementary References

1. Kim, H. & Yin, J. Effects of RNA splicing and post-transcriptional regulation on HIV-1 growth: a quantitative and integrated perspective. *Syst Biol* **152**, 138–152 (2005).

2. Reddy, B. & Yin, J. Quantitative intracellular kinetics of HIV type 1. *AIDS research and human retroviruses* **15**, 273–283 (1999).

3. Bohan, C. A. *et al.* Analysis of Tat transactivation of human immunodeficiency virus transcription in vitro. *Gene expression* **2**, 391–407 (1992).

4. Graeble, M. A., Churcher, M. J., Lowe, A. D., Gait, M. J. & Karn, J. Human immunodeficiency virus type 1 transactivator protein, tat, stimulates transcriptional read-through of distal terminator sequences in vitro. *Proc Natl Acad Sci U S A* **90**, 6184–6188 (1993).

5. Laspia, M. F., Wendel, P. & Mathews, M. B. HIV-1 Tat overcomes inefficient transcriptional elongation in vitro. (1993).

6. Wang, Y. & Lai, L. Modeling the intracellular dynamics for Vif-APO mediated HIV-1 virus infection. *Chinese Science Bulletin* **55**, 2329–2340 (2010).

7. Blanchard, J. M., Weber, J., Darnell, J. E. & Jelinek, W. In vitro RNA-RNA splicing in adenovirus 2 mRNA formation. *Proceedings of the National Academy of Sciences* **75**, 5344–5348 (1978).

8. Love, D. C., Sweitzer, T. D. & Hanover, J. A. Reconstitution of HIV-1 rev nuclear export: independent requirements for nuclear import and export. *Proc Natl Acad Sci U S A* **95**, 10608–10613 (1998).

9. Boyle, J. Molecular biology of the cell, 5th edition by B. Alberts, A. Johnson, J. Lewis, M. Raff, K. Roberts, and P. Walter. *Biochemistry and Molecular Biology Education* **36**, 317–318 (2008). arXiv:1011.1669v3.

10. Efthymiadis, A., Briggs, L. J. & Jans, D. A. The HIV-1 tat nuclear localization sequence confers novel nuclear import properties. *Journal of Biological Chemistry* **273**, 1623–1628 (1998).

11. Mahalingam, S. *et al.* Identification of residues in the N-terminal acidic domain of HIV-1 Vpr essential for virion incorporation. *Virology* **207**, 297–302 (1995).

12. Briggs, J. a. G. *et al.* The stoichiometry of Gag protein in HIV-1. *Nature structural & molecular biology* **11**, 672–675 (2004).

13. Streeck, H. *et al.* Antigen load and viral sequence diversification determine the functional profile of HIV-1-specific CD8+ T cells. *PLoS Medicine* **5**, 0790–0803 (2008).

14. Boutwell, C. L., Rowley, C. F. & Essex, M. Reduced Viral Replication Capacity of Human Immunodeficiency Virus Type 1 Subtype C Caused by Cytotoxic-T-Lymphocyte Escape Mutations in HLA-B57 Epitopes of Capsid Protein. *Journal of Virology* **83**, 2460–2468 (2009).

15. Kaul, R. *et al.* CD8+ lymphocytes respond to different HIV epitopes in seronegative and infected subjects. *The Journal of Clinical Investigation* **107**, 1303–1310 (2001).

16. Borghans, J. A. M., Mølgaard, A., de Boer, R. J. & Keşmir, C. HLA alleles associated with slow progression to AIDS truly prefer to present HIV-1 p24. *PLoS ONE* **2** (2007).

17. Kiepiela, P. *et al.* Dominant influence of HLA-B in mediating the potential co-evolution of HIV and HLA. *Nature* **432**, 769–75 (2004).

18. Matthews, P. C. *et al.* Central Role of Reverting Mutations in HLA Associations with Human Immunodeficiency Virus Set Point. *Journal of Virology* **82**, 8548–8559 (2008).

19. Dalchau, N. *et al.* A peptide filtering relation quantifies MHC class I peptide optimization. *PLoS Computational Biology* **7** (2011).
